# Temperature, pH, and oxygen availability contributed to the functional differentiation of ancient *Nitrososphaeria*

**DOI:** 10.1093/ismejo/wrad031

**Published:** 2024-01-10

**Authors:** Zhen-Hao Luo, Qi Li, Yuan-Guo Xie, Ai-Ping Lv, Yan-Ling Qi, Meng-Meng Li, Yan-Ni Qu, Ze-Tao Liu, Yu-Xian Li, Yang-Zhi Rao, Jian-Yu Jiao, Lan Liu, Manik Prabhu Narsing Rao, Brian P Hedlund, Paul N Evans, Yuan Fang, Wen-Sheng Shu, Li-Nan Huang, Wen-Jun Li, Zheng-Shuang Hua

**Affiliations:** State Key Laboratory of Biocontrol, Guangdong Provincial Key Laboratory of Plant Resources and Southern Marine Science and Engineering Guangdong Laboratory (Zhuhai), School of Life Sciences, Sun Yat-Sen University, Guangzhou 510275, PR China; State Key Laboratory of Biocontrol, Guangdong Provincial Key Laboratory of Plant Resources and Southern Marine Science and Engineering Guangdong Laboratory (Zhuhai), School of Life Sciences, Sun Yat-Sen University, Guangzhou 510275, PR China; Chinese Academy of Sciences, Key Laboratory of Urban Pollutant Conversion, Department of Environmental Science and Engineering, University of Science and Technology of China, Hefei 230026, PR China; State Key Laboratory of Biocontrol, Guangdong Provincial Key Laboratory of Plant Resources and Southern Marine Science and Engineering Guangdong Laboratory (Zhuhai), School of Life Sciences, Sun Yat-Sen University, Guangzhou 510275, PR China; Chinese Academy of Sciences, Key Laboratory of Urban Pollutant Conversion, Department of Environmental Science and Engineering, University of Science and Technology of China, Hefei 230026, PR China; State Key Laboratory of Biocontrol, Guangdong Provincial Key Laboratory of Plant Resources and Southern Marine Science and Engineering Guangdong Laboratory (Zhuhai), School of Life Sciences, Sun Yat-Sen University, Guangzhou 510275, PR China; State Key Laboratory of Biocontrol, Guangdong Provincial Key Laboratory of Plant Resources and Southern Marine Science and Engineering Guangdong Laboratory (Zhuhai), School of Life Sciences, Sun Yat-Sen University, Guangzhou 510275, PR China; State Key Laboratory of Biocontrol, Guangdong Provincial Key Laboratory of Plant Resources and Southern Marine Science and Engineering Guangdong Laboratory (Zhuhai), School of Life Sciences, Sun Yat-Sen University, Guangzhou 510275, PR China; Chinese Academy of Sciences, Key Laboratory of Urban Pollutant Conversion, Department of Environmental Science and Engineering, University of Science and Technology of China, Hefei 230026, PR China; Chinese Academy of Sciences, Key Laboratory of Urban Pollutant Conversion, Department of Environmental Science and Engineering, University of Science and Technology of China, Hefei 230026, PR China; State Key Laboratory of Biocontrol, Guangdong Provincial Key Laboratory of Plant Resources and Southern Marine Science and Engineering Guangdong Laboratory (Zhuhai), School of Life Sciences, Sun Yat-Sen University, Guangzhou 510275, PR China; State Key Laboratory of Biocontrol, Guangdong Provincial Key Laboratory of Plant Resources and Southern Marine Science and Engineering Guangdong Laboratory (Zhuhai), School of Life Sciences, Sun Yat-Sen University, Guangzhou 510275, PR China; Instituto de Ciencias Aplicadas, Facultad de Ingeniería, Universidad Autónoma de Chile, Sede Talca, 3460000 Talca, Chile; School of Life Sciences, University of Nevada Las Vegas, Las Vegas, NV 89154, United States; Nevada Institute of Personalized Medicine, University of Nevada Las Vegas, Las Vegas, NV 89154, United States; The Australian Centre for Ecogenomics, School of Chemistry and Molecular Biosciences, University of Queensland, St Lucia, QLD 4072, Australia; Chinese Academy of Sciences, Key Laboratory of Urban Pollutant Conversion, Department of Environmental Science and Engineering, University of Science and Technology of China, Hefei 230026, PR China; Institute of Ecological Science, Guangzhou Key Laboratory of Subtropical Biodiversity and Biomonitoring, Guangdong Provincial Key Laboratory of Biotechnology for Plant Development, School of Life Sciences, South China Normal University, Guangzhou 510631, PR China; Guangdong Provincial Key Laboratory of Chemical Pollution, South China Normal University, Guangzhou 510006, PR China; State Key Laboratory of Biocontrol, Guangdong Provincial Key Laboratory of Plant Resources and Southern Marine Science and Engineering Guangdong Laboratory (Zhuhai), School of Life Sciences, Sun Yat-Sen University, Guangzhou 510275, PR China; State Key Laboratory of Biocontrol, Guangdong Provincial Key Laboratory of Plant Resources and Southern Marine Science and Engineering Guangdong Laboratory (Zhuhai), School of Life Sciences, Sun Yat-Sen University, Guangzhou 510275, PR China; State Key Laboratory of Desert and Oasis Ecology, Key Laboratory of Ecological Safety and Sustainable Development in Arid Lands, Xinjiang Institute of Ecology and Geography, Chinese Academy of Sciences, Urumqi 830011, PR China; Chinese Academy of Sciences, Key Laboratory of Urban Pollutant Conversion, Department of Environmental Science and Engineering, University of Science and Technology of China, Hefei 230026, PR China

**Keywords:** *Nitrososphaeria*, functional differentiation, archaeal evolution, metagenomics, acidophile, thermophile

## Abstract

Ammonia-oxidizing *Nitrososphaeria* are among the most abundant archaea on Earth and have profound impacts on the biogeochemical cycles of carbon and nitrogen. In contrast to these well-studied ammonia-oxidizing archaea (AOA), deep-branching non-AOA within this class remain poorly characterized because of a low number of genome representatives. Here, we reconstructed 128 *Nitrososphaeria* metagenome-assembled genomes from acid mine drainage and hot spring sediment metagenomes. Comparative genomics revealed that extant non-AOA are functionally diverse, with capacity for carbon fixation, carbon monoxide oxidation, methanogenesis, and respiratory pathways including oxygen, nitrate, sulfur, or sulfate, as potential terminal electron acceptors. Despite their diverse anaerobic pathways, evolutionary history inference suggested that the common ancestor of *Nitrososphaeria* was likely an aerobic thermophile. We further surmise that the functional differentiation of *Nitrososphaeria* was primarily shaped by oxygen, pH, and temperature, with the acquisition of pathways for carbon, nitrogen, and sulfur metabolism. Our study provides a more holistic and less biased understanding of the diversity, ecology, and deep evolution of the globally abundant *Nitrososphaeria*.

## Introduction

Since *Nitrososphaeria* (synonym *Thaumarchaeota*) was first detected by 16S rRNA gene surveys in marine water columns [1, 2], they have gained considerable attention due to their profound impacts on the biogeochemical cycling of carbon and nitrogen in most environments [3-6]. It is now recognized that this class is metabolically diverse, widely distributed, and massively abundant in oceans and soils, so its evolutionary origin, early evolution, and impact on the paleoecology of terrestrial and marine systems are of great interest [7-11].

All currently known ammonia-oxidizing archaea (AOA) fall within the order *Nitrososphaerales*, with recent genomic and paleolipid data linking their distribution and diversification to major climatic events such as glaciation, greenhouse climates, and deep-ocean oxygenation [12-14]. It is reported that gene duplication [15] and transfer [16] are important to the evolution of *Nitrososphaeria*. However, these previous studies have only included a few genomes outside of the *Nitrososphaerales* and disagree on the ancestral traits [17] and the timing of the evolutionary origin of AOA [12, 13]. Informal names have been given to several deeply rooted lineages within the *Nitrososphaeria*, such as Group I.1c [18], pSL12 group [19], and pSL12-like group [20] (hereafter referred to together as non-AOA), which are so far known only through 16S rRNA gene sequences or metagenome-assembled genomes (MAGs). Besides the common absence of genes for ammonia oxidation, different terminal oxidases for respiring oxygen were revealed in some deep-branching non-AOA [21]. However, due to the limited number and diversity of MAGs, the physiology and ecology of these extant non-AOA and the early evolution of *Nitrososphaeria* have not been comprehensively studied.

Extreme environments with harsh physicochemical conditions imposing challenges to life are hot spots for discoveries of novel archaea [22], including poorly studied lineages within the *Nitrososphaeria*. In particular, *Nitrososphaeria* MAGs have previously been recovered from acid mine drainage (AMD) environments [23, 24], where they are likely to be the sole ammonia oxidizers. Since pH has been suggested as a major driver of niche diversification in *Nitrososphaeria* [25], the origin and evolution of these presumably acidophilic *Nitrososphaeria* are of special interest. Additionally, geothermal springs harbor diverse groups of non-AOA [21, 26], including the only isolate *Conexivisphaera calidus*, a strict anaerobe with the capacity for sulfur and iron reduction [26], and were likely to be the habitats where the common ancestor of *Nitrososphaeria* arose [19, 27, 28]. Therefore, AMD and geothermal springs are both important ecosystems to study the ecology and evolution of the ancestors of the *Nitrososphaeria* as a whole.

To gain deeper insights into the ecological roles of non-AOA and the deep evolutionary history of *Nitrososphaeria*, we reconstructed 128 new *Nitrososphaeria* MAGs, mostly identified as non-AOA, from 45 AMD and hot spring sediments, and investigated their functional potential within the context of existing *Nitrososphaeria* genomes. The expanded set of non-AOA *Nitrososphaeria* is metabolically diverse, with annotated pathways for carbon fixation via the Wood–Ljungdahl pathway (WLP); respiration with oxygen, nitrate, sulfur, or sulfate as terminal electron acceptors; carbon monoxide oxidation; and methanogenesis. Combined with their wide distribution in extreme environments, these non-AOA may influence the biogeochemical cycles of carbon, nitrogen, and sulfur in those environments. Evolutionary genomic analyses suggested that the common ancestor of *Nitrososphaeria* may have been aerobic and originated in thermal habitats. Available oxygen, temperature, and pH played vital roles in the evolution of *Nitrososphaeria*, but different orders were shaped by different factors, which led to their functional divergence. Our analysis provides a much-needed critical analysis of the likely physiology and ecology of *Nitrososphaeria* and the factors shaping their functional diversification in their various environments.

## Materials and methods

### Sampling, DNA extraction, and metagenomics sequencing

A total of 36 AMD sediment samples were collected from 19 mine tailings sites across 4 provinces (Anhui, Guangdong, Guangxi, and Jiangxi) located in the south of China in July and August 2017. The physicochemical properties were measured as previously described [29]. For pH measurement, 4.0-g sediment samples were mixed with 10 ml deionized water and was measured with a pH meter. These samples were acidic with high concentrations of sulfate and heavy metals (see Supplementary Tables S1 and S2, Supplementary Fig. S1 for detailed geographical and physicochemical parameters). We obtained nine hot spring sediment samples from Tengchong, Yunnan, China. Details regarding sample collection and DNA preparation for metagenomic sequencing were described in previous studies [19, 30]. All samples were collected in 50-ml tubes (sterile) and stored in liquid nitrogen. Then, they were immediately transported to the lab and stored long-term at −80°C. DNA extraction was performed with a FastDNA Spin Kit (MP Biomedicals, Irvine, CA, USA) under the manufacturer’s protocol. Libraries were prepared with the NEB Next Ultra DNA Library Prep Kit for Illumina (New England Biolabs, Beverly, MA, USA), and metagenomic sequencing data were generated on the HiSeq2500 platform (2 × 150 bp).

### Metagenome assembly and genome binning

Raw sequences were processed to remove duplicated and low-quality reads as described previously [31]. The high-quality reads of each sample were assembled using SPAdes v3.14.0 [32] using a range of k-mers (*k* = 21, 33, 55, 77, 99, 127) under the meta mode. Read mapping was conducted using BBMap v.36.77 (https://sourceforge.net/projects/bbmap/) with the parameters “minid = 0.97, local=t” to determine the abundance and coverage of scaffolds (length ≥ 2500 bp). Then, combined with the sequence composition, the abundance (or coverage) information of scaffolds was used by MetaBAT v2.12.1 [33], MaxBin v2.2.2 [34], Abawaca v1.00 (https://github.com/CK7/abawaca), and Concoct v0.4.0 [35] with default parameters to the initial binning results. These bins were de-replicated and optimized via DASTools v.1.0.0 [36] and manually curated via RefineM v0.24.0 [37]. Completeness and contamination of MAGs were evaluated using CheckM v1.0.7 [38] under the “lineage_wf” option. To obtain MAGs with the optimal quality, the “reassemble bins” module in MetaWRAP v1.3.2 [39] was applied and the final binning set was selected according to the quality of origin and reassembled MAGs. Then, reference genomes of *Nitrososphaeria* were downloaded from NCBI database (Supplementary Data S1, final data collection in July 2021). Only genomes with completeness ≥50% and contamination <10% were kept for downstream analysis.

### Functional annotation and ecological distribution investigation

Prodigal v2.6.3 [40] with the “-p single” option was employed to predict the open reading frames (ORFs) of all MAGs. Then, the ORFs were annotated against the KEGG [41] and arCOG [42] databases using DIAMOND v2.0.6 [43] with E-values <1e-5. Protein domains were annotated with InterProScan v5.55 [44]. The metabolic profiles of each MAGs were summarized by the abovementioned result to produce a count table (Supplementary Data S2). Assignments of key metabolic pathways and specific functions were manually verified based on the count table and the KEGG mapper (https://www.genome.jp/kegg/mapper.html).

The relative abundance of each MAG was estimated by the fraction of metagenomic reads assigned to each MAG [45] (Supplementary Data S3). The global distribution and abundance of *Nitrososphaeria* were evaluated with the Integrated Microbial Next Generation Sequencing (IMNGS) [46] server at a 97% similarity threshold and minimal length of 100 bp. In brief, all 16S rRNA gene surveys in Short Read Archive datasets (SRA; www.ncbi.nlm.nih.gov/sra) were screened for closely related 16S rRNA sequences extracted in this study. To minimize the risk of false positives, we retained only those cases where matched reads >1 in the corresponding amplicon sequencing data. Metadata of the respective SRA datasets was provided in NCBI. The habitat categories were manually curated by the habitat information.

### Phylogenetic and phylogenomic analyses

The 54 concatenated ribosomal proteins [47] (Supplementary Table S3) were chosen to generate phylogenetic trees of *Nitrososphaeria*, which each of them was shared by more than 50% of all genomes in this study. Multiple sequence alignments of the individual ribosomal proteins were built using MUSCLE v3.8.31 [48] with default parameters. Poorly aligned regions were removed using TrimAL v1.4 [49] with the parameters “-gt 0.05 –cons 50”. The maximum-likelihood phylogeny for the concatenated alignment was inferred using the IQ-TREE v2.1.3 [50] with 1000 UFBoot replicates [51]. The best-fitting protein model, Q.yeast+R10, was determined using ModelFinder [52].

The 16S rRNA gene sequences were identified using SSU-ALIGN v.0.1.1 (http://eddylab.org/software/ssu-align/) and aligned on the SILVA web interface (https://www.arb-silva.de) through the SINA alignment algorithm. The filtration of alignment was applied with TrimAL to eliminate poorly aligned regions. The 16S rRNA gene tree was inferred using IQ-TREE as described above, and SYM + R6 was chosen as the best substitution model. In terms of other genes of interest, see Supplementary figure legends for detailed phylogeny reconstruction. All trees were uploaded to iTOL [53] for visualization.

### Comparative genomics analyses and ancestral trait reconstruction

Optimal growth temperatures (OGTs) for genomes with completeness ≥80% and contamination ≤5% were predicted using frequencies of seven amino acids (IVYWREL) [54]. Significant correlation was observed between the predicted and experimentally determined OGTs (Supplementary Fig. S2, *R*^2^ = 0.8106, *P* < .001), indicating the reliability of OGT prediction in this study. Ancestral OGTs were retrieved with fastAnc function and the visualized with contMap function in phytools package (v1.5-1). Principle coordinate analysis (PCoA) and adonis analysis were conducted using the KEGG profiles of each MAG with vegan package (v2.6-4) in R. Data visualizations were obtained with ggplot2 package (v3.3.5). The respiration types were inferred from the CoxAB (aerobic) and CydAB (microaerobic), and the habitat types were inferred from the source descriptions of the genomes.

### Gene tree-aware ancestral reconstruction

As the accuracy of ancestral reconstruction could be influenced by the genome completeness, only the abovementioned genomes with completeness ≥80% and contamination ≤5% abovementioned were selected as the final dataset. Protein families were defined based on aforementioned functional annotation results. Sequences that belonged to the same KOs/arCOGs/IPRs were extracted. Only gene families with ≥4 sequences and each with length ≥ 30 amino acids were retained for subsequent analysis. Within each family, sequences were aligned using MUSCLE, followed by processing with TrimAL and IQ-TREE utilizing the same parameters as mentioned above. In terms of *coxAB*, sequences from AOA group were clustered at a threshold of 0.95, considering that the majority of AOA genomes contain *coxAB* genes. The uneven distribution of this complex across the entire *Nitrososphaeria* may impede the precise reconstruction of its evolutionary history. Manual filtrations were carried out to eliminate the alignment region of *coxC* with BioEdit [55], resulting from the fusion of C-terminus of *coxA* to the *coxC* gene in Archaea [56]. Protein families of interest were reconciled against the supermatrix tree (outgroup genomes were also included) constructed based on 54 concatenated ribosomal proteins using the ALEml_undated algorithm (sampling 100 times) of the ALE package v1.0. This approach allowed us to infer the numbers of intra-LGT (HGTs occurred among sampled genomes), duplications, losses, and originations (gene birth or HGTs from outside the studied taxonomic group of organisms) on each branch of the supermatrix tree [57]. During the analysis, genome completeness evaluated by CheckM was used as input to avoid the biased estimation of evolutionary events caused by the incomplete genomes. The generated outputs were processed using in-home script (https://github.com/hzhengsh/ALE_result_parser). We specifically applied a frequency threshold of 0.3 to identify events, accounting for potential noise that may arise from sequence alignment and tree reconstructions. This step was crucial to ensure that positive signals were not obscured by such noise. The potential horizontally transferred genes were further validated by phylogenetic analyses.

## Results and discussion

### Expanded phylogenetic diversity of *Nitrososphaeria*

**Figure 1:**
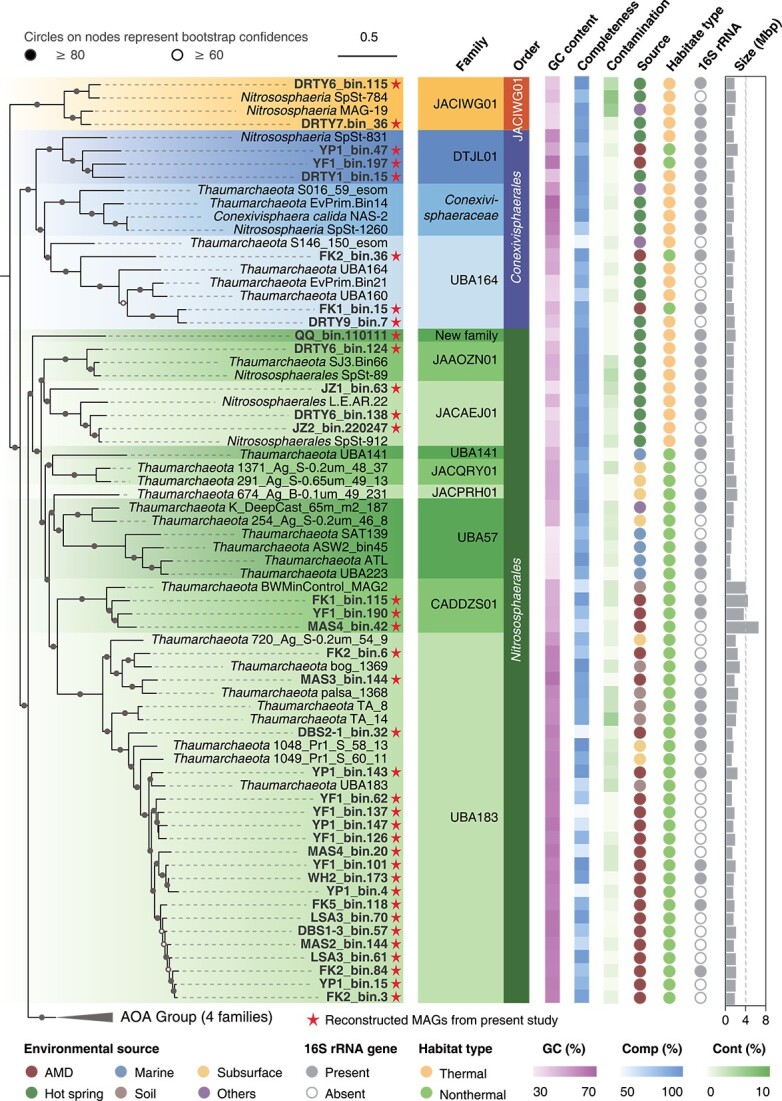
Phylogenetic placement and genomic characteristics of *Nitrososphaeria*. Maximum likelihood phylogenomic tree of *Nitrososphaeria* was constructed based on a concatenation of 54 markers (8862 columns, see Materials and Methods for details). Six genomes from *Nitrososphaeria_A*, *Thermoprotei_A*, and *Korarchaeia* were chosen as outgroups. AOA families are collapsed to emphasize non-AOA that comprise 13 of the 17 families. The family-level classification was provided by colored shading. Nodes with ultrafast bootstrap value ≥ 80% (60%) were indicated as solid (hollow) circles. The scale bar in the middle indicates 50% sequence divergence.

A total of 128 *Nitrososphaeria* MAGs including 105 from AMD and 23 from hot spring sediments were reconstructed in the present study. Twenty-eight of the MAGs were determined to be high quality, with the remaining classified as medium quality [58]. Nine MAGs were abundant (>1%) in their source communities with relative abundances up to 3.8% (Supplementary Data S3). An additional 456 reference genomes from NCBI were combined with these new MAGs and then de-replicated at 95% average nucleotide identity, resulting in 274 species-level representative genomes with high average completeness (86.2% ± 0.9%) and low contamination (1.6% ± 0.1%). The extrapolated genome sizes range from 0.8 to 6.5 Mbp (median: 1.5 Mbp). These MAGs encode an average of 1, 892 protein-coding sequences, and the mean gene length is 718 bp. A maximum likelihood phylogenetic tree inferred from the concatenation of 54 single-copy proteins provided a well-supported topology with bootstrap confidences of most internal nodes >80% (Fig. 1). Approximately 64% (176) of all genomes in the full genome set contain 16S rRNA genes (Supplementary Data S1 and S3), and the topology of the 16S rRNA gene phylogeny was broadly congruent with the corresponding phylogenomic tree (Supplementary Fig. S3). According to the relative evolutionary distance as implemented in GDTB-Tk, the full diversity of available *Nitrososphaeria* genomes could be assigned to 3 orders and 17 families [59], with 13 families of non-AOA represented and the remainder being AOA lineages. The newly assembled MAGs from AMD and hot spring sediments were classified into eight non-AOA and two AOA families, leading to a notable increase of diversity within existing *Nitrososphaeria* lineages. In particular, 20 representative genomes, obtained through the de-replication of 79 MAGs exclusively reconstructed from AMD sediments (Supplementary Data S3), were classified within the family UBA183 (Fig. 1). Five and four additional MAGs were, respectively, assigned to DTJL01 and CADDZS01, each of which contained only one representative genome prior to the current study. One MAG, QQ_bin.110111, which was recovered from hot spring sediments, represents a new family. To our knowledge, the functional characteristics of 7 of the 13 non-AOA families remain unstudied, with less than four genomes each. By searching 16S rRNA gene sequences extracted from these MAGs against IMNGS database, results showed that these families are primarily distributed in soils, freshwater, marine, and hypersaline habitats (Supplementary Data 4). Overall, our study has increased the available non-AOA *Nitrososphaeria* genomes from 63 to 175. This substantial increase in genome diversity provided a great opportunity to gain insight into the ecology and evolution of this group. Our genome-resolved metagenomics revealed that non-AOA are more phylogenetically diverse than their well-studied, ammonia-oxidizing counterparts (13 non-AOA families within three orders versus four AOA families belonging to a single order).

We also found, for the first time, putative AOA (LSA2_bin.105) in AMD environments with pH values <3, as evidenced by the identification of ammonia monooxygenase encoded by *amoABC* (Supplementary Fig. S4). This was unexpected since the documented lowest pH for the growth of AOA in pure culture was 4.0 [60] and is surprising as ammonia, with pKa of ammonia/ammonium, which is 9.25 at 25°C, would be scarce. We recognize that comparing environmental samples to pure cultures introduces complexity. Nonetheless, we believe that this observation serves as a strong incentive for future research endeavors to uncover how these microorganisms thrive and whether they retain the capacity for ammonia oxidation under such an inhospitable environment.

### Metabolic diversity of non-AOA

**Figure 2:**
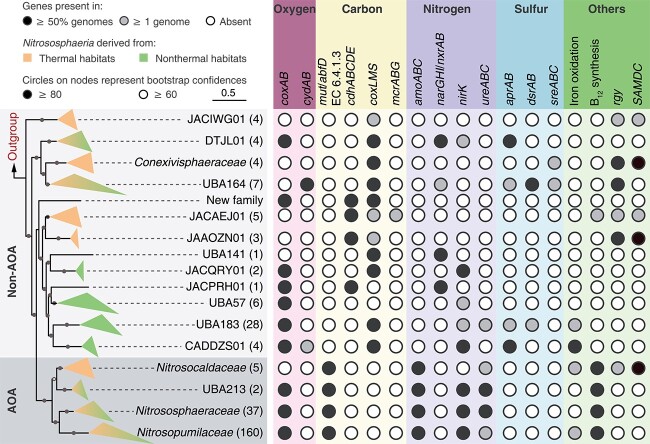
The overall metabolic potentials of *Nitrososphaeria*. The phylogenomic tree on the left was collapsed according to Figure 1. For visualization and simplicity, genomes belonging to the same family were collapsed (AOA-Group were shaded with grey). Numbers in parentheses described the number of genomes in the corresponding family. Circles represented the occurrence frequency of genes/complexes in a given lineage. The absence of the circles indicated the absence of the pathways (genes). HP/HB cycle, hydroxypropionate/hydroxybutyrate cycle; *coxAB*, cytochrome c oxidase; *cydAB*, cytochrome bd ubiquinol oxidase; *mut*, methylmalonyl-CoA mutase; *abfD*, 4-hydroxybutyryl-CoA dehydratase/vinylacetyl-CoA-delta-isomerase; EC 6.4.1.3, biotin-dependent acetyl-CoA/propionyl-CoA carboxylase; *cdhABCDE*, CODH/ACS complex; *coxSML*, carbon monoxide dehydrogenase; *mcrABG*, methyl-coenzyme M reductase; *amoABC*, ammonia monooxygenase; *narGHI/nxrAB*, nitrate reductase/nitrite oxidoreductase; *nirK*, nitrite reductase; *ureABC*, urease; *aprAB*, adenylylsulfate reductase; *dsrAB*, dissimilatory sulfite reductase; *sreABC*, sulfur reductase; *rgy*, reverse gyrase; SAMDC, *S*-adenosylmethionine decarboxylase/arginine decarboxylase.

Genomes from non-AOA *Nitrososphaeria* are not only phylogenetically diverse but also functionally divergent. Two variants of respiratory Complex IV were detected in *Nitrososphaeria* genomes, namely Type A heme-copper oxygen reductases (HCOs) encoded by *coxAB* that are adapted to high levels of oxygen and the higher affinity cytochrome *bd* oxidases encoded by *cydAB* that are better adapted to low concentration of oxygen. Save for families JACIWG01, JACAEJ01, and UBA141, all *Nitrososphaeria* harbor at least one type of Complex IV (Fig. 2 and Supplementary Fig. S5). *coxAB* genes were more prevalent than *cydAB* genes among genomes derived from both thermal and non-thermal habitats, suggesting the broad distribution of aerobic lifestyles across the lineage. Both *coxAB* and *cydAB* genes were detected in one MAG within family CADDZS01, potentially allowing adaptation to a wide range of oxygen levels and microhabitats. Only five MAGs in families UBA164 and CADDZS01 encode both *cydA* and *cydB* genes, suggesting that they are microaerophiles, with most remaining MAGs lacking the *cydB* gene, which is associated with oxygen binding [61]. We observed a general trend that most *coxAB*-encoding non-AOA are derived from non-thermal habitats, while most thermophiles encode only *cydAB* or neither *coxAB* nor *cydAB*. This result is plausible given the relatively lower oxygen solubility in hot water [62].

In contrast to AOA genomes, several families of non-AOA, including JACPRH01 from non-thermal habitats, and JACAEJ01 and JAAOZN01, and the new family represented by QQ bin_110 111, all from thermal environments, harbor a complete tetrahydromethanopterin (H_4_MPT)-dependent WLP. This pathway may allow them to fix carbon dioxide, based on the presence of genes for the key enzyme carbon monoxide dehydrogenase/acetyl-CoA synthase (CdhABCDE, Fig. 2 and Supplementary Fig. S5) and the complete archaeal methyl-branch of the WLP (Supplementary Data S2). Remarkably, the MAG JZ2_bin.220247 harbors the *mcr* complex, along with genes for methyltransferases and corrinoid proteins (*mtaA* and *mttBC*) (Supplementary Data S2), possibly conferring a capacity for H_2_-dependent methylotrophic methanogenesis, as reported previously [30]. Carbon monoxide could be exploited via the aerobic carbon monoxide dehydrogenase (*coxLMS*) complex, which is broadly distributed in non-AOA families but completely absent in AOA. Phylogenetic analysis showed that all *cox* complexes within non-AOA were exclusively classified as Form II (Supplementary Fig. S6), which have a lower affinity to CO compared to Form I *cox* complexes, potentially conferring non-AOA with physiological advantages in environments with high CO concentrations [63, 64].

Unexpectedly, a few anaerobic *Nitrososphaeria* MAGs encode *coxLMS* complexes, which are typically involved in aerobic respiration. We speculate that these microorganisms likely use nitrate rather than oxygen as the electron acceptor during CO oxidation [63, 65]. This inference could be exemplified by the detection of *narGHI* genes among anaerobic *Nitrososphaeria*, including families DTJL01, UBA164, and UBA141 (Fig. 2 and Supplementary Fig. S5). The patchy distribution of *narG* genes from *Nitrososphaeria* suggested possible horizontal acquisitions. Phylogenetic analysis revealed that *narG* was likely endowed by bacteria via two independent horizontal gene transfers (HGTs) (Supplementary Fig. S7). Two types of *narG* genes were detected, with sequences from QQ_bin.110111 and *Nitrososphaeria* archaeon NC_groundwater_674_Ag_B-0.1μm_49_231 belonging to the periplasmic type, and sequences from YP1_bin.47, DRTY9_bin.7 and DRTY1_bin.15 belonging to the cytoplasmic type, revealing niche differentiation among *Nitrososphaeria* presumably caused by the different affinities of these enzymes for nitrate [66] and their different free energy yields [67, 68]. Homologs of NirK were also detected in nine non-AOA genomes interspersed within five families, including CADDZS01, DTJL01, JACQRY01, UBA57, and UBA183, and phylogenetic analysis suggested that they were also acquired from bacteria in three independent HGT events (Supplementary Fig. S8).

To date, only one MAG from non-AOA *Nitrososphaeria* has been previously reported to contain dissimilatory sulfite reductase genes (*dsrAB*), which would enable dissimilatory reduction of sulfite to sulfide [69]. In the present study, DsrAB homologs were annotated in six additional non-AOA genomes, with two belonging to family UBA183 and four belonging to UBA164 (Fig. 2 and Supplementary Fig. S5). The *aprAB*, *sat*, and *dsrMKOP* genes were detected in most MAGs, suggesting a likely complete reduction of sulfate to sulfide (Supplementary Data S2). We hypothesize that these putative *Nitrososphaeria* sulfate reducers likely thrive in AMD environments because sulfate is abundant in these environments. None of these MAGs encode *dsrD* (Supplementary Fig. S9), an allosteric activator of DsrAB [70]. The presence of genes for maturation factor *dsrR* and the sulfur relay genes *dsrCEFH*, indicators of sulfur oxidation [71], in YP1_bin.143 suggests sulfur oxidation may also be possible (Supplementary Fig. S9). In addition to the *dsr* complex, the presence of sulfur reductase encoded by *sre* suggests that UBA164 could be capable of reducing S^0^ and S_2_O_3_^−^ as well [26]. Rusticyanin, encoded by *rus*, was detected in four families, including the common AMD families UBA183 and CADDZS01, and *Nitrosocaldaceae* and *Nitrosopumilaceae* derived mostly from geothermal and marine environments, suggesting the capacity for iron oxidation and also further reinforces the high potential metabolic diversity of *Nitrososphaeria* lineages (Fig. 2 and Supplementary Fig. S5).

### Temperature, pH, and oxygen contributed to the functional diversification within *Nitrososphaeria*

**Figure 3:**
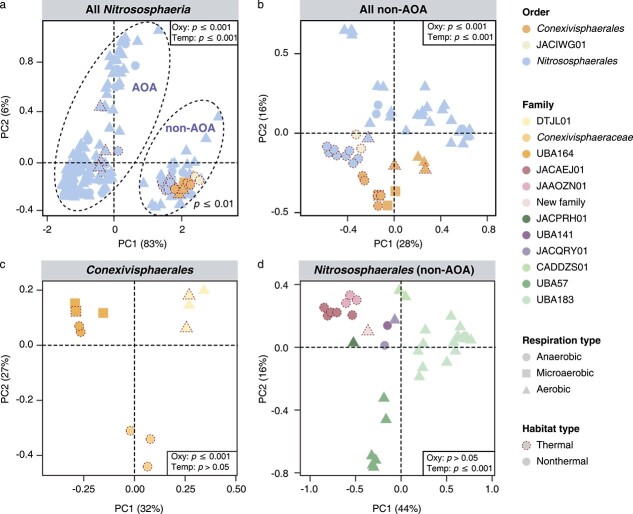
Genomic comparisons between different *Nitrososphaeria* clades. (A–D) Principal coordinate analysis (PCoA) plots with Bray–Curtis distances based on the functional profiling of all *Nitrososphaeria* genomes (A), all non-AOA genomes (B), genomes from *Conexivisphaerales* (C), and from non-AOA of *Nitrososphaerales* (D) annotated by the KEGG database. Different line types represented the type of the source environment (dashed: thermal; solid: mesothermal), and shapes represented whether it could potentially adopt an aerobic lifestyle (triangle: aerobic (*coxAB* detection); square: microaerobic (*cydAB* detection); circle: anaerobic). The analysis of similarity (ADONIS) statistics considers genomes from different groups.

Against a background of limited genomic diversity [15, 72, 73], a previous study of a single *amoA* gene revealed that pH adaptation likely led to the niche expansion of *Nitrososphaeria* [25]. Here, with a wider taxonomic range of *Nitrososphaeria* lineages encompassing a greater number of genes, a more comprehensive comparative genomic analysis was performed to evaluate factors that led to the diversification of *Nitrososphaeria* lineages. Principal coordinates analysis (PCoA) based on KOs segregated AOA and non-AOA into two distinct clusters (Fig. 3A). Such clear separation indicates that environmental factors, physiological, and evolutionary responses to those drivers may have resulted in remarkable functional differentiation between AOA and non-AOA. Oxygen availability has been suggested to be an important driver of the differentiation of *Nitrososphaeria* during the transition between anaerobic and aerobic respiration [12]. As *Nitrososphaeria* inhabit a wide range of thermal habitats, we also examined whether temperature of the sampling sites where MAGs were recovered correlates with genome contents. The results showed that both temperature and oxygen correlate with the gene composition of this class (Fig. 3A and B, Adonis, *P* values <.001), which is concordant with a previous study suggesting habitat drove the diversification of *Nitrososphaeria* [19]. Consistent with the inference that pH is important for the diversification of AOA [25], our analysis showed that pH was also important when expanded to the non-AOA group (PERMANOVA: pseudo-F = 2.6, *R*^2^ = 0.05, *P* = .002). However, individual orders seem to be primarily shaped by differing factors. For instance, temperature (PERMANOVA: pseudo-F = 1.0, *R*^2^ = 0.06, *P* = .4) and pH (PERMANOVA: pseudo-F = 0.6, *R*^2^ = 0.04, *P* = .9) do not correlate with the gene content of *Conexivisphaerales* (Fig. 1); instead, oxygen seems to contribute more to their genomic variations (Fig. 3C). Specifically, all genomes within this order form three clusters according to their phylogenetic position. Members of family DTJL01 are entirely aerobes based on the presence of *coxAB* genes, while all microorganisms in *Conexivisphaeraceae* are anaerobes as neither *coxAB* nor *cydAB* were detected (Supplementary Fig. S5). UBA164 are likely microaerophiles due to the ubiquity of *cydAB*, which exhibits a high affinity for oxygen. Phylogenetic analysis revealed that frequent HGTs occurred among *Nitrososphaeria*, *Thermoplasmatota*, and *Micrarchaeota* (Supplementary Fig. S10). Given the absence of genes encoding the oxygen-binding subunit CydB in a few *Conexivisphaeraceae* genomes, we reasoned that these microorganisms are likely anaerobes. However, we cannot rule out the possibility that genome incompleteness may have led to the absence of this complex in certain lineages. In contrast, temperature (Fig. 3D) and pH (PERMANOVA: pseudo-F = 2.2415, *R*^2^ = 0.06, *P* = .015), rather than oxygen, correlated with genomic differences of non-AOA within *Nitrosophaerales*. While genomic variations within the order JACIWG01 do not correlate with oxygen, temperature, or pH, this is likely because there are too few genome representatives in this clade.

**Figure 4:**
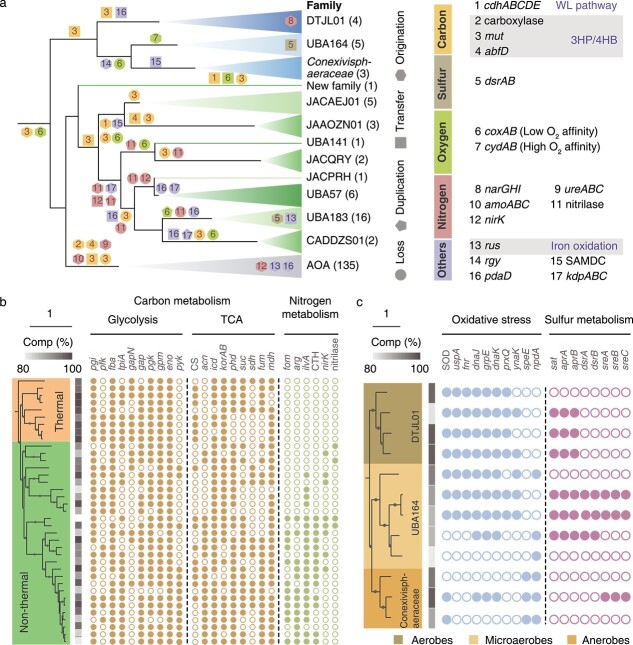
Evolutionary history reconstruction of *Nitrososphaeria*. (A) Summary of ancestral gene events along the evolution of *Nitrososphaeria*. The taxonomic groups were distinguished using the same color scheme as shown in Figure 1. The numerical values and colors on each node correspond to genes and belonging functional categories (carbon, nitrogen, sulfur, etc.), as listed on the right side of the figure. Different shapes are employed where numbers are present to indicate the gene gain (through HGT, duplication, or origination) or loss events. The comparisons of genes of interest between members from different groups: (B) thermophiles and non-thermophiles in non-AOA of *Nitrososphaerales*; (C) aerobes, microaerophiles, and anaerobes in *Conexivisphaerales*. Due to the lack of representative genomes, JACIWG01 was excluded in the evolutionary analysis. *cdhABCDE*, CODH/ACS complex; *mut*, methylmalonyl-CoA mutase; *abfD*, 4-hydroxybutyryl-CoA dehydratase/vinylacetyl-CoA-Delta-isomerase; *dsrAB*, dissimilatory sulfite reductase; *coxAB*, cytochrome c oxidase; *cydAB*, cytochrome bd ubiquinol oxidase; *narGHI*, nitrate reductase; *ureABC*, urease; *amoABC*, ammonia monooxygenase; *nirK*, nitrite reductase (NO-forming); *rus*, rusticyanin; *rgy*, reverse gyrase; SAMDC, *S*-adenosylmethionine decarboxylase/arginine decarboxylase; *pdaD*, arginine decarboxylase; *kdpABC*, potassium-transporting ATPase; *pgi*, glucose-6-phosphate isomerase; *pfk*, phosphofructokinase; *fba*, fructose-bisphosphate aldolase; *tpiA*, triosephosphate isomerase; *gap*, glyceraldehyde-3-phosphate dehydrogenase; *pgk*, phosphoglycerate kinase; *gpm*, phosphoglycerate mutase; *eno*, enolase; *pyk*, pyruvate kinase; CS, citrate synthase; *acn*, aconitate hydratase; *icd*, isocitrate dehydrogenase; *korAB*, 2-oxoglutarate/2-oxoacid ferredoxin oxidoreductase; *phd*, dihydrolipoamide dehydrogenase; *suc*, succinyl-CoA synthetase; *sdh*, succinate dehydrogenase; *fum*, fumarate hydratase; *mdh*, malate dehydrogenase; *fom*, formamidase; *arg*, arginase; *ilvA*, threonine dehydratase; CTH, cystathionine gamma-lyase; SOD, superoxide dismutase; *uspA*, universal stress protein A; *fnr*, ferredoxin/flavodoxin—NADP^+^ reductase; *dnaJ*, molecular chaperone DnaJ; *GrpE*, molecular chaperone GrpE; *dnaK*, molecular chaperone DnaK; *prxQ*, peroxiredoxin; *ynaK*, redox-sensitive bicupin protein; *speE*, spermidine synthase; *npdA*, NAD-dependent protein deacetylase; *sat*, sulfate adenylyltransferase.

To better understand the influences of temperature, pH, and oxygen in shaping the gene content of *Nitrososphaeria*, the evolutionary history of 17 key genes/complexes involved in carbon, nitrogen, sulfur, and oxygen metabolism, as well as adaptations to thermal and acidic conditions, was reconstructed (Fig. 4A). A total of 1080 evolutionary events were identified, made up of 478 acquisitions and 602 losses. Among the acquisition events, 454 (95.0%) were HGTs (within the species tree), illustrating the crucial role in gain of function leading to niche differentiation. Save for the deepest branching order JACIWG01, aerobic respiration is likely to be an ancient feature, as evidenced by an origination event of *coxAB* at the common ancestor of the two orders *Conexivisphaerales* and *Nitrososphaerales* (Fig. 4A). Despite frequent HGT events observed across the entire class, we speculate that the *coxAB* genes from AOA, DTJL01, and CADDZS01 might have been inherited from their common ancestor (Supplementary Fig. S11). However, the evolutionary history of this complex could be intricate, and other possibilities cannot be ruled out. A similar evolutionary scenario has also been observed in *Thermoplasmatota*, where the *coxAB* genes in this phylum have undergone frequent and independent acquisitions via HGT [74]. Considering the common presence of *coxAB* genes in thermophiles, such as *Sulfolobus acidocaldarius* [56], it is unsurprised to assume that the thermophilic ancestor of *Conexivisphaerales* and *Nitrososphaerales* may have possessed the *coxAB* complex. It has been reported that the solubility of O_2_ in water at 70°C is ~60% compared to that at 25°C [75]. The predicted OGT (53.7–64.2°C) suggests that the common ancestor exhibited moderate thermophily, indicating an environment with potentially higher O_2_ solubility. Furthermore, the common ancestor of *Nitrososphaeria* is estimated to date back to 2.6 billion years ago [12], slightly predating the great oxidation event (~2.3 billion years ago). Therefore, the evolution of the common ancestor of two families likely occurred later in a context where oxygen was already present, propelling the evolution of aerobic respiration. Collectively, these lines of evidence support the possibility that there is a considerable amount of oxygen available to promote the evolution of *coxAB* in the common ancestor of *Conexivisphaerales* and *Nitrososphaerales*. In concert with previous studies [17, 19, 27, 28], we inferred a hot origin of *Nitrososphaeria* and AOA since the predicted OGTs of both ancestral states were high (54.2–66.6°C and 45.6–56.1°C; Supplementary Fig. S12) and several lineages derived from thermal habitats located deep within the phylogeny (Fig. 1). This includes nearly all members in the JACIWG01 and *Conexivisphaerales* lineages. Moreover, the three families that branched deeply within the *Nitrososphaerales* including QQ_bin.110111, JAAOZN01, and JACAEJ01 were also derived from thermal environments. The wide distribution of revere gyrase [76-78], encoded by *rgy*, further suggested that these families are thermophilic or hyperthermophilic and are consistent with habitat temperatures greater than 60°C and a mean predicted OGT of 65.0°C (Supplementary Data S1 and S3). Ancestral state reconstruction confirmed the inference that the *rgy* gene was present at the common ancestor of *Conexivisphaeraceae* and UBA164, though frequent HGTs occurred (Fig. 4A; Supplementary Fig. S13). In addition, *S*-adenosylmethionine decarboxylase, encoded by SAMDC, which is involved in polyamine production, was likely present at the common ancestor of JACAEJ01 and JAAOZN01, indicating an additional strategy to cope with high-temperature stress [79]. The hot-origin could be extended to their closest neighbors, *Caldarchaeales* (formerly known as *Aigarchaeota*), as the common ancestor of *Caldarchaeales* and *Nitrososphaeria* shared similar gene contents with the common ancestor of *Nitrososphaeria* [19].

Adaptation to oxygen may have triggered genomic divergence of the *Conexivisphaerales* (Fig. 3C). Given the presence of *coxAB* at the ancestral node of orders *Conexivisphaerales* and *Nitrososphaerales*, we reasoned that their common ancestor was likely aerobic and originated from oxic environments. The prevalence of an aerobic carbon monoxide dehydrogenase (*coxSML*) among both orders supports this inference (Supplementary Fig. S5), although phylogenetic analysis of the CoxL homologs indicated that frequent HGTs have occurred (Supplementary Fig. S6). Aerobic microorganisms experience reactive oxygen species and require enzymes to cope with oxidative stress. As expected, MAGs from the aerobic family DTJL01 and the microaerophilic family UBA164 encode many enzymes to combat oxidative stress, in contrast to the obligate anaerobes in the *Conexivisphaeraceae* (Fig. 4C). We also observed an apparent transition with regard to sulfur metabolism. Both sulfite and sulfur reductases, encoded by *dsrAB* and *sreABC* genes, coexisted with *cydAB* complexes in several MAGs, suggesting that these microorganisms might be facultatively anaerobic microaerophiles. Phylogenetic analysis of *dsrAB* genes placed *Nitrososphaeria* homologs adjacent to the genus *Vulcanisaeta* and *Pyrobaculum* within the reductive archaeal clade (Supplementary Fig. S14 and Supplementary Data 5), implying potential genetic exchange between *Nitrososphaeria* and *Thermoprotei.* The phylogeny of *sre* revealed that this complex in UBA164 may have been transferred horizontally from bacteria and then transferred again to *Conexivisphaeraceae* (Supplementary Fig. S15). While the evolution of *aprAB* complex was also affected by oxygen, its evolutionary trajectory differs from *dsrAB* and *sreABC* complexes as organisms containing this complex are exclusively from either strict aerobes or facultative anaerobes.

Apart from the three deep branching families (QQ_bin.110111, JAAOZN01, and JACAEJ01) within the non-AOA in the *Nitrososphaerales*, all remaining families in this order seem to exclusively inhabit environments with lower temperatures as suggested by their predicted OGTs (Supplementary Fig. S16 and Supplementary Data S1, and S2). This result consolidates the inference [19] that temperature likely played a crucial role in the diversification of these microorganisms (Fig. 1). Despite the hot origin of the order (predicted OGT: 53–62°C, Supplementary Fig. S12), the presence of *coxAB* at the ancestral node of the *Nitrososphaerales* suggests that the common ancestor of the order may have been aerobic and evolved in oxic environments (Fig. 4A). A subsequent gene loss event occurred at the common ancestor of the families JACAEJ01 and JAAOZN01, revealing a transition to an anaerobic lifestyle in these two families. Without exception, all microorganisms from the three families branching deepest in the *Nitrososphaerales* possess the key genes involved in the WLP, *cdhABCDE*, suggesting its antiquity (Fig. 2 and Supplementary Fig. S3). In addition to the HP/HB cycle in AOA, the WLP in these thermophilic non-AOA is an alternative autotrophic pathway within the *Nitrososphaeria*. The presence of both *coxAB* and *cdhABCDE* in QQ_bin.1110111 suggests the potential for this MAG to be a facultative anaerobe since the latter is highly sensitive to oxygen [80, 81]*.* No implicit evidence supported the HGT of *cdh* complex in QQ_bin.1110111 (Supplementary Fig. S17). Instead, they may be the donor for previous acquisitions by *Asgardarchaeota* according to *Cdh* phylogeny. Compelling evidence demonstrated that the *cdh* complexes in families JACAEJ01 and JAAOZN01 might be inherited vertically. This is congruent with the evolutionary inference that the origination event of *cdh* occurred at the common ancestor of JACAEJ01 and JAAOZN01 rather than entire order (Fig. 4A). The phylogeny of formylmethanofuran dehydrogenases encoded by *fwdABCD*, another key complex involved in the WLP, reveals a similar evolutionary history as *cdh*; that is, most target genes in *Nitrososphaeria* are adjacent to *Bathyarchaeia* except for QQ_bin.1110111, which might have been obtained horizontally (Supplementary Fig. S18). Combined with the vertical inheritance of one copy of *coxAB* (the second copy was horizontally acquired), we infer an ancestral nature of aerobic respiration and acquired capacity of carbon fixation in QQ_bin.1110111. The detection of both complexes reflects a high degree of genomic plasticity in non-AOA *Nitrososphaeria*, enabling these lineages to respond rapidly to changes and diversify into different niches. This flexibility is further exemplified by the presence of a *mcrABG* complex in one MAG in JACAEJ01, suggesting that they occupy a methanogenic niche.

As the *Nitrososphaeria* expanded into environments with a lower temperature at the last common ancestor of families UBA141, JACQRY01, JACPRH01, UBA57, UBA183, and CADDZS01, they evolved capacity to utilize and tolerate oxygen based on the presence of *coxAB* genes in most genomes. As anticipated, all but one genome lost the oxygen-sensitive WLP. Surprisingly, *cydAB* genes are much less prevalent in these MAGs, suggesting that high oxygen is favorable. They also simultaneously gained genes involved in nitrogen transformations, including homologs of formamidase, nitrate reductase (NirK), nitrilase, arginase (Arg), threonine dehydratase (IlvA), and cystathionine gamma-lyase (CTH), equipping them with the capacity for nitrogen turnover (Fig. 4B).

As expected, two nodes (the common ancestor of family UBA141, JACQRY01, JACPRH01, UBA57, UBA183, and CADDZS01) and the common ancestor of the latter four families at which ancestral non-AOA expanded into acidic environments corresponded to the acquisition of homologs of arginine decarboxylase (PdaD) and potassium-transporting ATPase (KdpABC) (Fig. 4A). PdaD and KdpABC are involved in the decarboxylation of arginine in the cytoplasm by consuming protons and generation of a reverse membrane potential by inhibiting proton influx [82]. Similar to the phylum *Thermoplasmatota*, both gene complexes coping with acid stress seem to be acquired via HGT rather than inherited from their common ancestor [74]. These and other enzyme systems such as a phosphate transport system (PstABCS), CPA1 family monovalent cation: H^+^ antiporter (TC. TPA1), SSS family solute: Na^+^ symporter (TC.SSS), and V-type ATPases [16] confer resistance in acidic environments are enriched for in non-AOA allowing them inhabit these environments (Supplementary Fig. S19).

## Conclusions

The phylogeny of *Nitrososphaeria* is dominated by deeply branching non-AOA whose ecology and evolution remain poorly understood. Benefitting from the high diversity of MAGs obtained from the AMD and geothermal environments, this study greatly expands our knowledge of the phylogenetic and functional diversity of non-AOA *Nitrososphaeria*, as well as their evolutionary past. Together with their wide distribution and probable roles in carbon, nitrogen, and sulfur cycles, these little-known archaea might have an underappreciated contribution to global biogeochemical cycles in the past. The significant functional disparities among different lineages were likely shaped by available oxygen, temperature, and pH. The driving forces are lineage-specific and different orders exhibit radically different evolutionary trajectories. While members of *Conexivisphaerales* were largely affected by oxygen availability, non-AOA within the *Nitrososphaerales* were primarily shaped by temperature. To adapt to new environments, non-AOA *Nitrososphaeria* evolved novel niches through HGT, acquiring the capacities for nitrate, sulfur, or sulfite reduction; carbon monoxide oxidation; and carbon fixation. This study represents a crucial first step toward resolving the distinct mechanisms leading to the genomic evolution and ecological success of *Nitrososphaeria* first in extreme environments and later as diverse and abundant AOA in many non-extreme biomes.

## Supplementary Material

Supplementary_Data_1_wrad031

Supplementary_Data_2_wrad031

Supplementary_Data_3_wrad031

Supplementary_Data_4_wrad031

Supplementary_Data_5_wrad031

Supplementary_Information_clean_wrad031

## Data Availability

The MAGs in this paper have been deposited to NCBI with the project accession number PRJNA666095 (accession numbers for each uploaded MAG was recorded in Supplementary Data S3).
