# Supplementary material for: Temperature, pH, and oxygen availability contributed to the functional differentiation of ancient Nitrososphaeria
Source: ISME J. 2024 Jan 10;18(1):wrad031. doi: 10.1093/ismejo/wrad031 (PMC10833072; doi:10.1093/ismejo/wrad031)
Supplement: Supplementary_Information_clean_wrad031 [file supplementary_information_clean_wrad031.docx]

Supplementary information

**Temperature, pH, and oxygen availability contributed to the functional differentiation of ancient *Nitrososphaeria***

Luo et al.

**The PDF file includes:**

Supplementary Figs. S1 to S19

Supplementary Tables S1 to S3

Legends for Supplementary Data 1 to 5

References

**Other Supplementary Materials for this manuscript includes the following:**

Supplementary Data 1 to 5


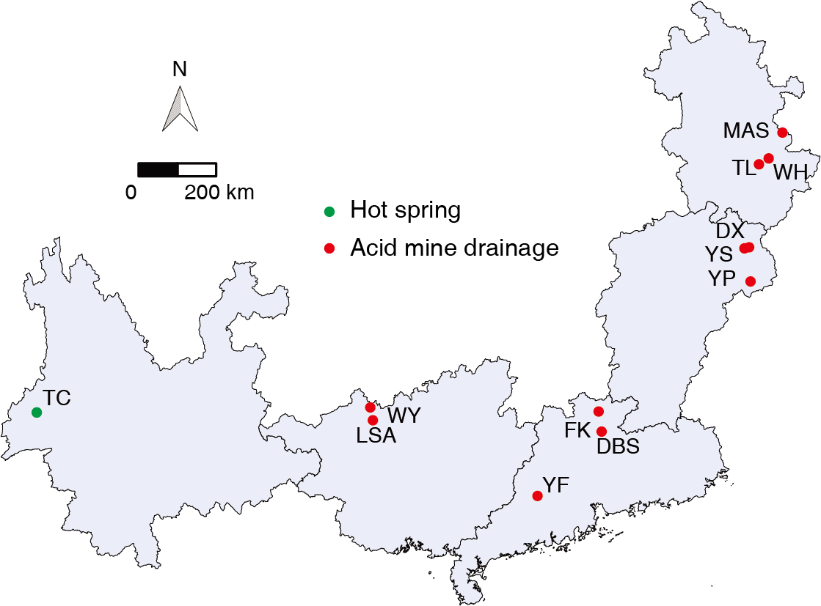


**Fig. S1 Location of sampling sites.** AMD samples were collected in 11 AMD tailings across Southeast China and hot spring sediment samples were collected from Tengchong County in Yunnan, China.


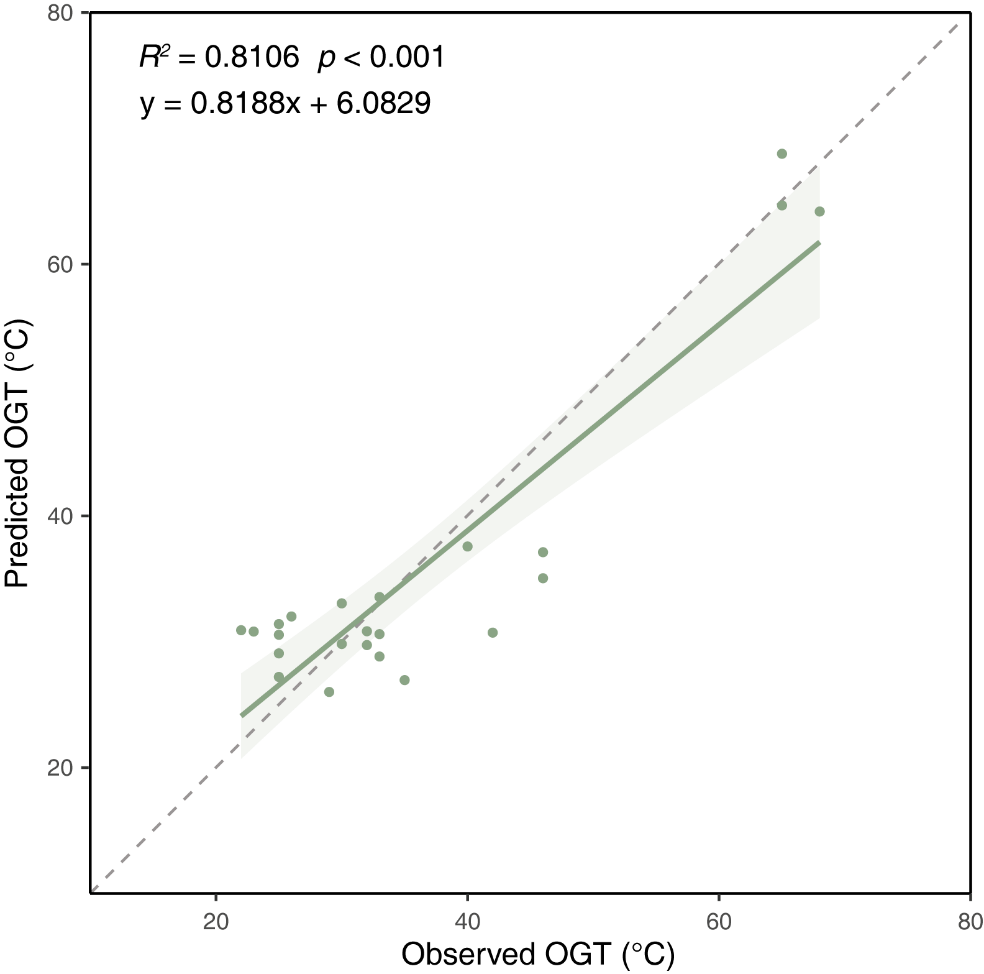


**Fig. S2 Linear regression between the predicted and experimentally determined optimal growth temperatures** (**OGTs).** Given the limited availability of pure cultures and their corresponding OGT data for microbes in *Nitrososphaeria*, the comparison was conducted where both types of data were available (data available in Supplementary Data 1).


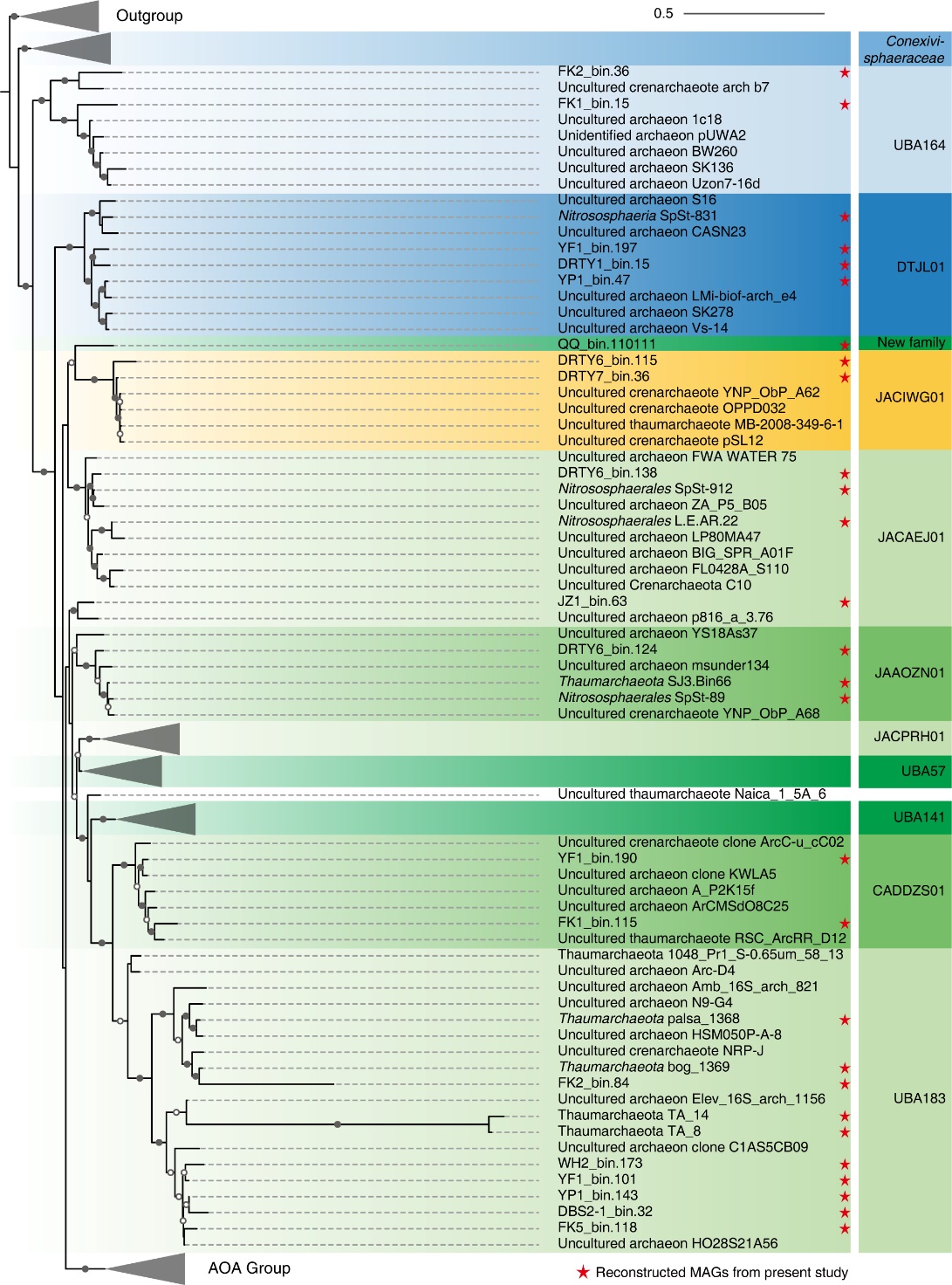


**Fig. S3** **Maximum-likelihood-based phylogenetic tree of 16S rRNA genes in *Nitrososphaeria*.** 16S rRNA gene sequences from *Nitrososphaeria_A*, *Thermoprotei_A*, and *Korarchaeia* were chosen as outgroups. An alignment length of 2, 942 columns was acquired and used for the phylogeny reconstruction (See Methods). Sequences retrieved from non-AOA MAGs were labeled in red. Nodes with ultrafast bootstrap values ≥ 80% (60%) were indicated as solid (hollow) circles.


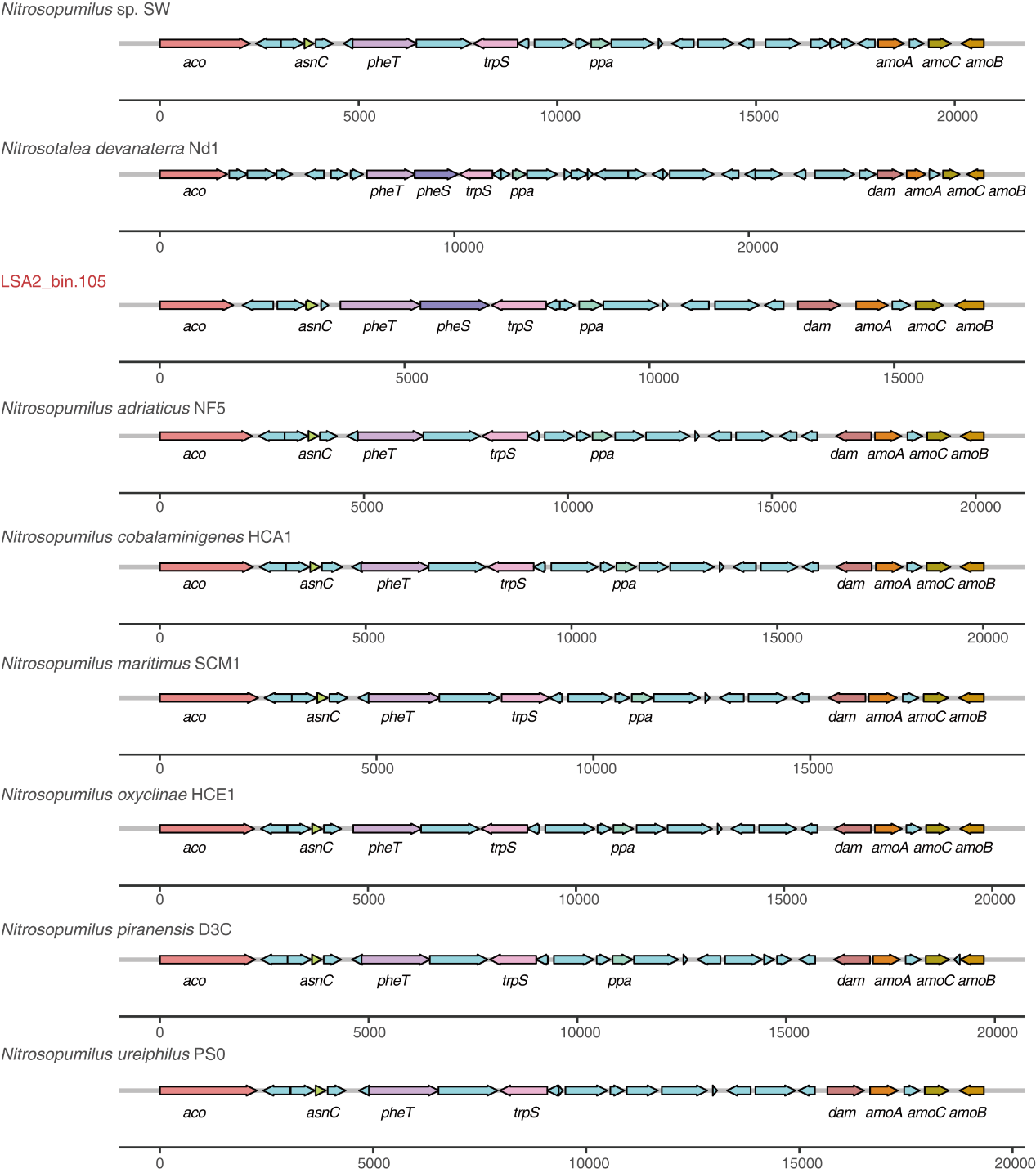


**Fig. S4** **The AmoACB operon cluster in AOA obtained from AMD environment in this study and previously reported AOA isolates.** Genome reconstructed in this study was marked in red. Colors represented different genes. Abbreviation: *aco*, aconitate hydratase; *asnC*, Lrp/AsnC family transcriptional regulator; *pheT*, phenylalanyl-tRNA synthetase beta chain; *pheS*, phenylalanyl-tRNA synthetase alpha chain; *trpS*, tryptophanyl-tRNA synthetase; *ppa*, inorganic pyrophosphatase; *dam*, DNA adenine methylase; *amoABC*, ammonia monooxidase.


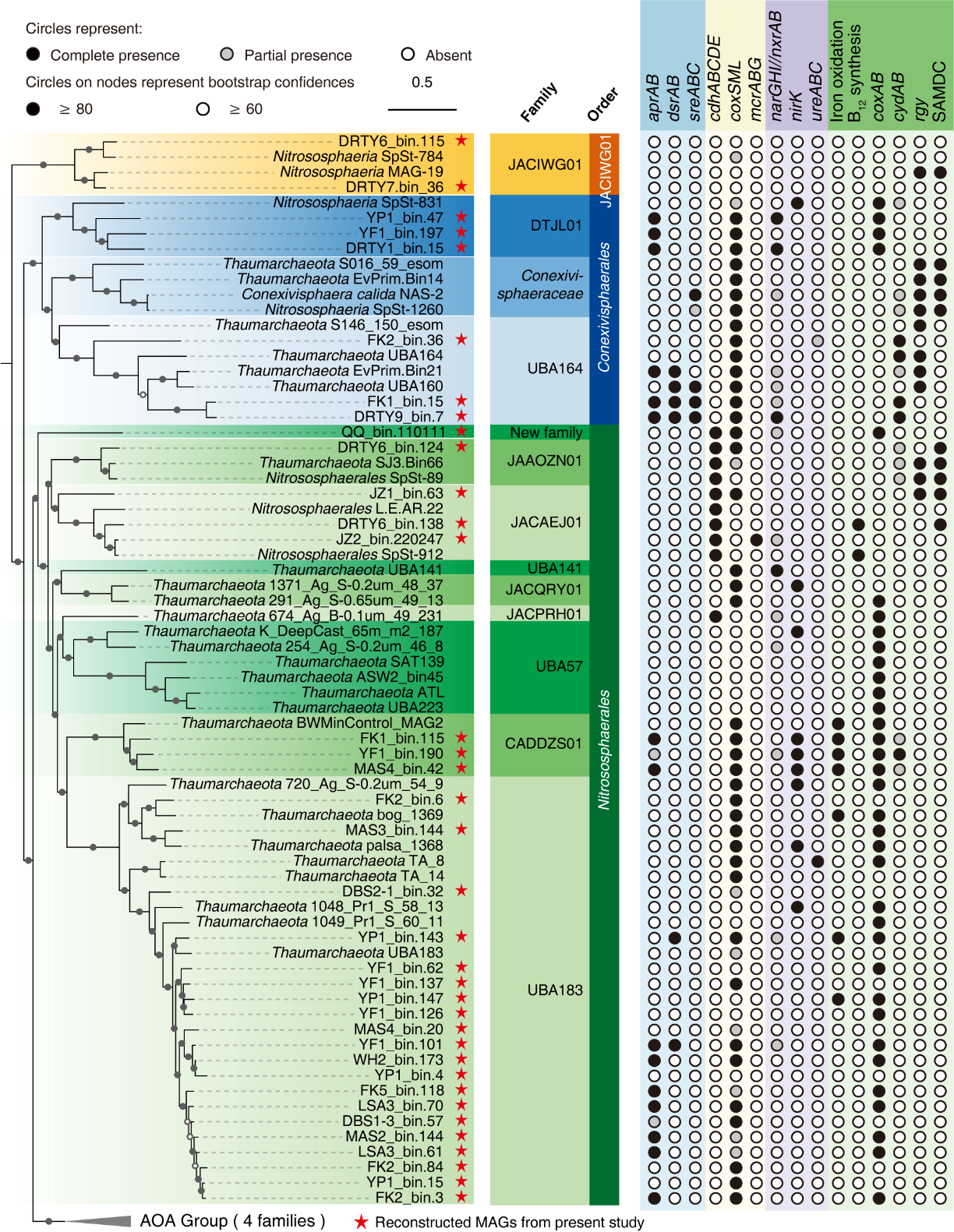


**Fig. S5** **The overall metabolic potentials of Non-AOA in *Nitrososphaeria*.** The phylogenomic tree shown on the left was the same as in Fig 1. The black, grey, and white circles at right indicated the complete presence, partial presence, and absence of the corresponding genes/complexes, respectively.
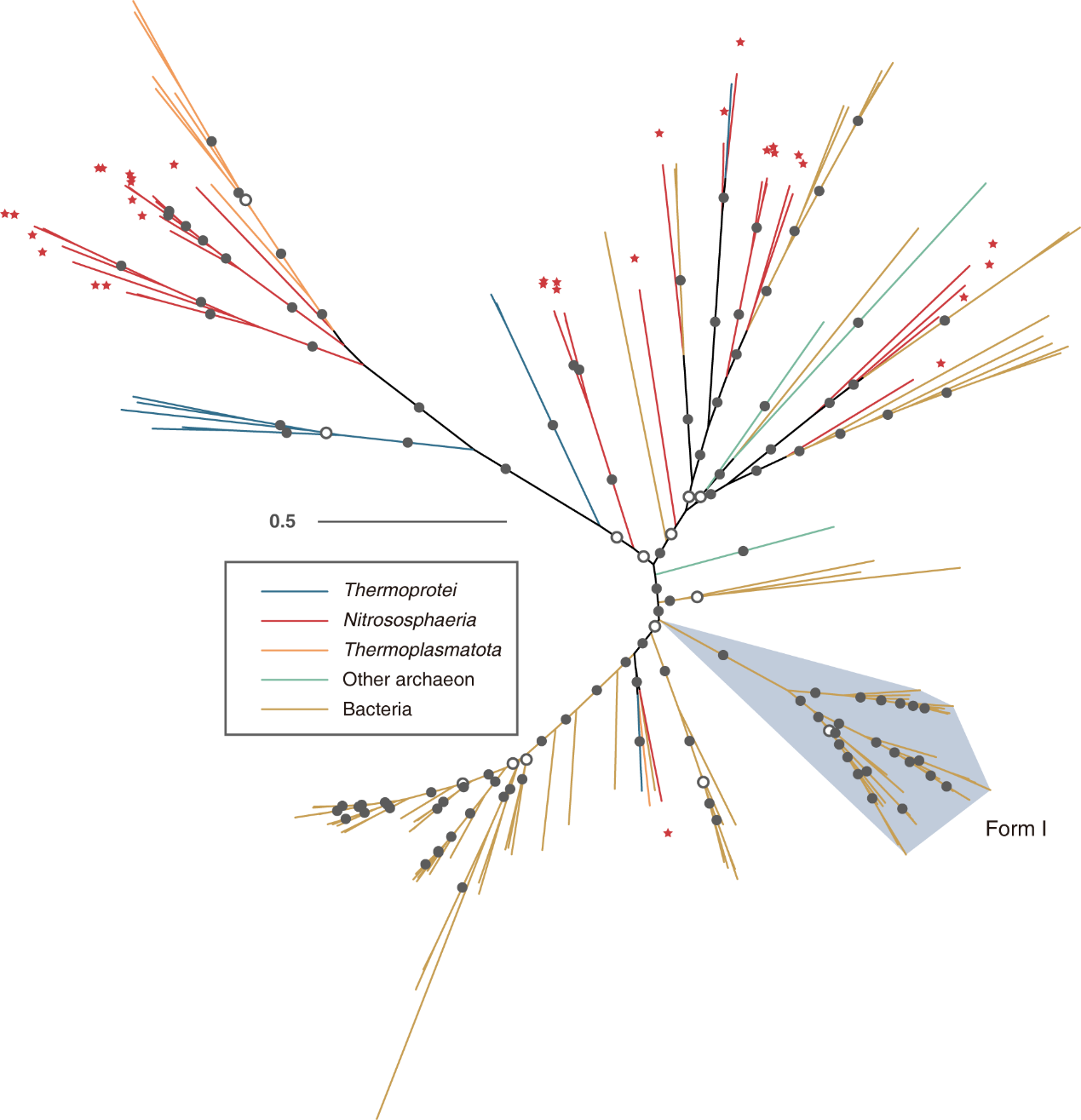


**Fig. S6 Maximum-likelihood phylogenetic tree of the homologs of CoxL.** Amino acid sequences from the tree were from King et al. [1, 2] and obtained from NCBI database. All sequences were aligned with MUSCLE v3.8.31 [3] and the alignments were filtered with TrimAL v1.4 [4], resulting in an alignment length of 626 columns. The phylogeny was inferred with IQ-TREE v2.1.3 [5] and LG+R7 was chosen as the best substitution model. Sequences from *Nitrososphaeria* were marked with red stars. Nodes with ultrafast bootstrap values ≥ 80% (60%) were indicated as solid (hollow) circles.


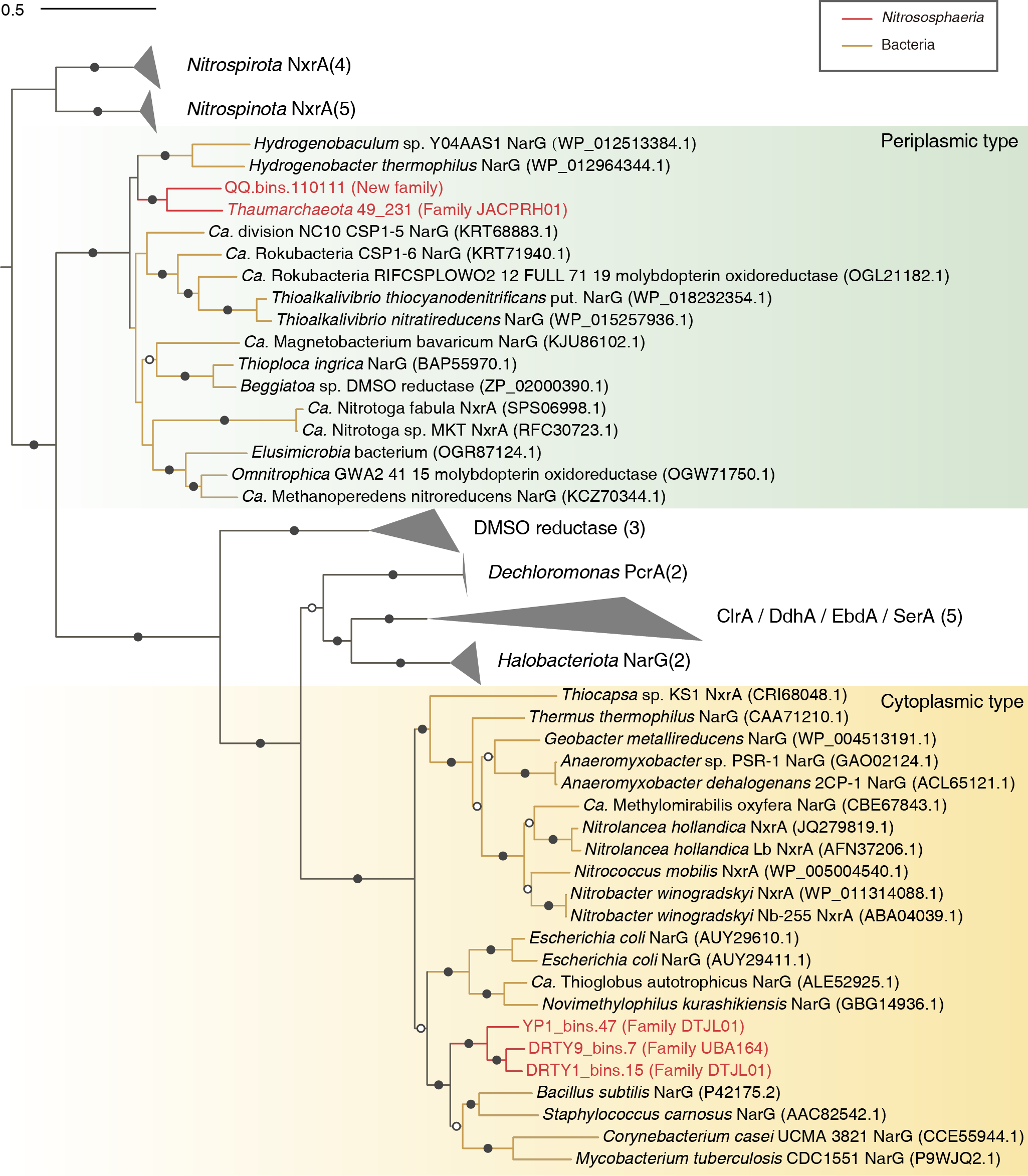


**Fig. S7** **Maximum-likelihood phylogenetic tree of the homologs of NarG.** Amino acid sequences from the tree were from Kitzinger et al. [6] and obtained from the NCBI database. All sequences were aligned with MUSCLE [3] and the alignments were filtered with TrimAL [4], resulting in an alignment length of 839 columns. The phylogeny was inferred with IQ-TREE [5] and LG+F+R4 was chosen as the best substitution model. Sequences from *Nitrososphaeria* were labeled in red. Nodes with ultrafast bootstrap values ≥ 80% (60%) were indicated as solid (hollow) circles.


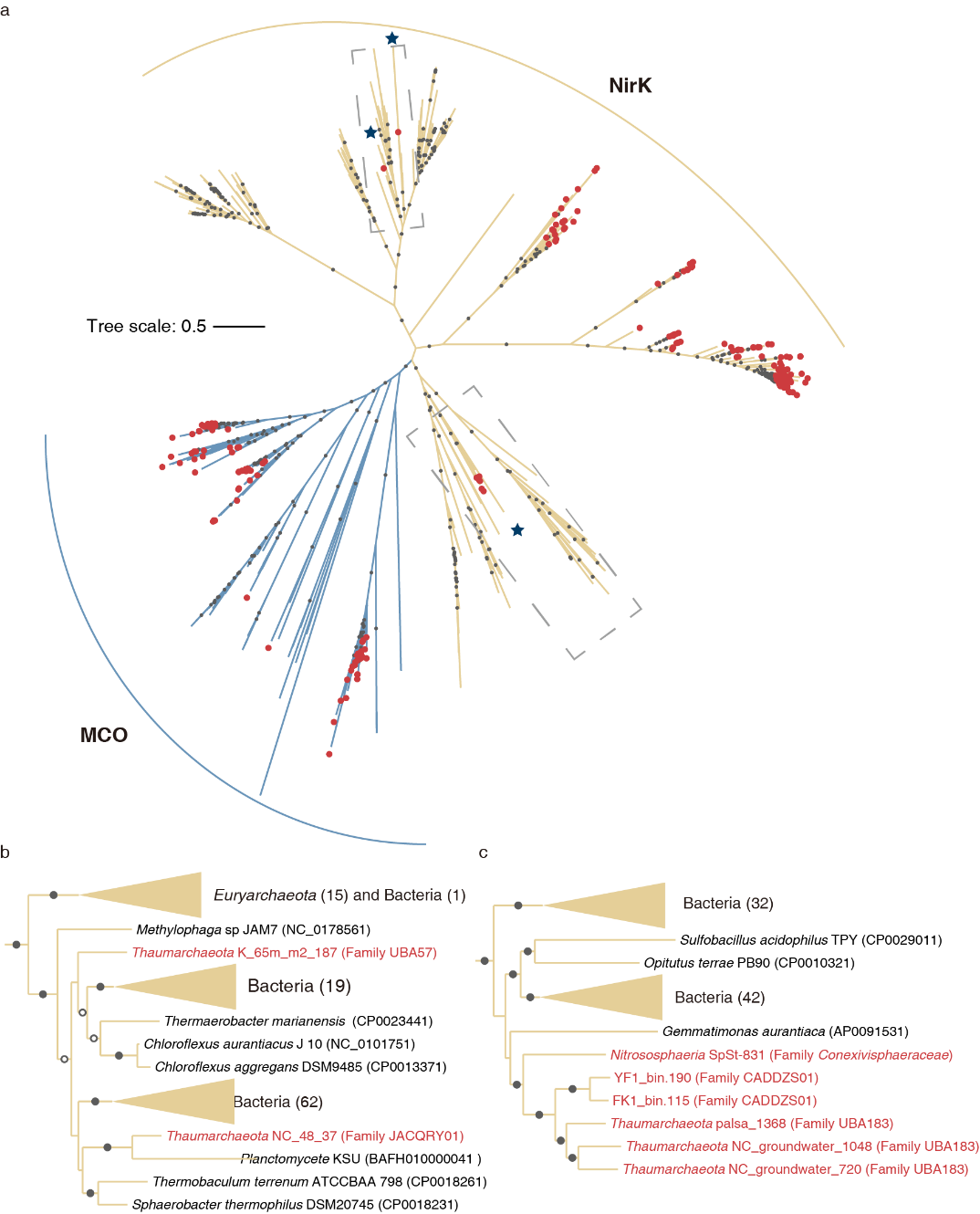


**Fig. S8** **Maximum-likelihood phylogenetic tree of the homologs of NirK.** Amino acid sequences from the tree were from Helen et al. [7] and Kerou et al. [8] and obtained from the NCBI database. All sequences were aligned with MAFFT v7.508 [9] and the alignments were filtered with TrimAL [4], resulting in an alignment length of 1, 438 columns. The phylogeny was inferred with IQ-TREE [5] and Q.pfam+R8 was chosen as the best substitution model. (a) Sequences from *Nitrososphaeria* were marked with red dots. (b-c) The enlarged phylogenies of lineages with dashed boxes labeled in (a). Nodes with ultrafast bootstrap values ≥ 80% (60%) were indicated as solid (hollow) circles.


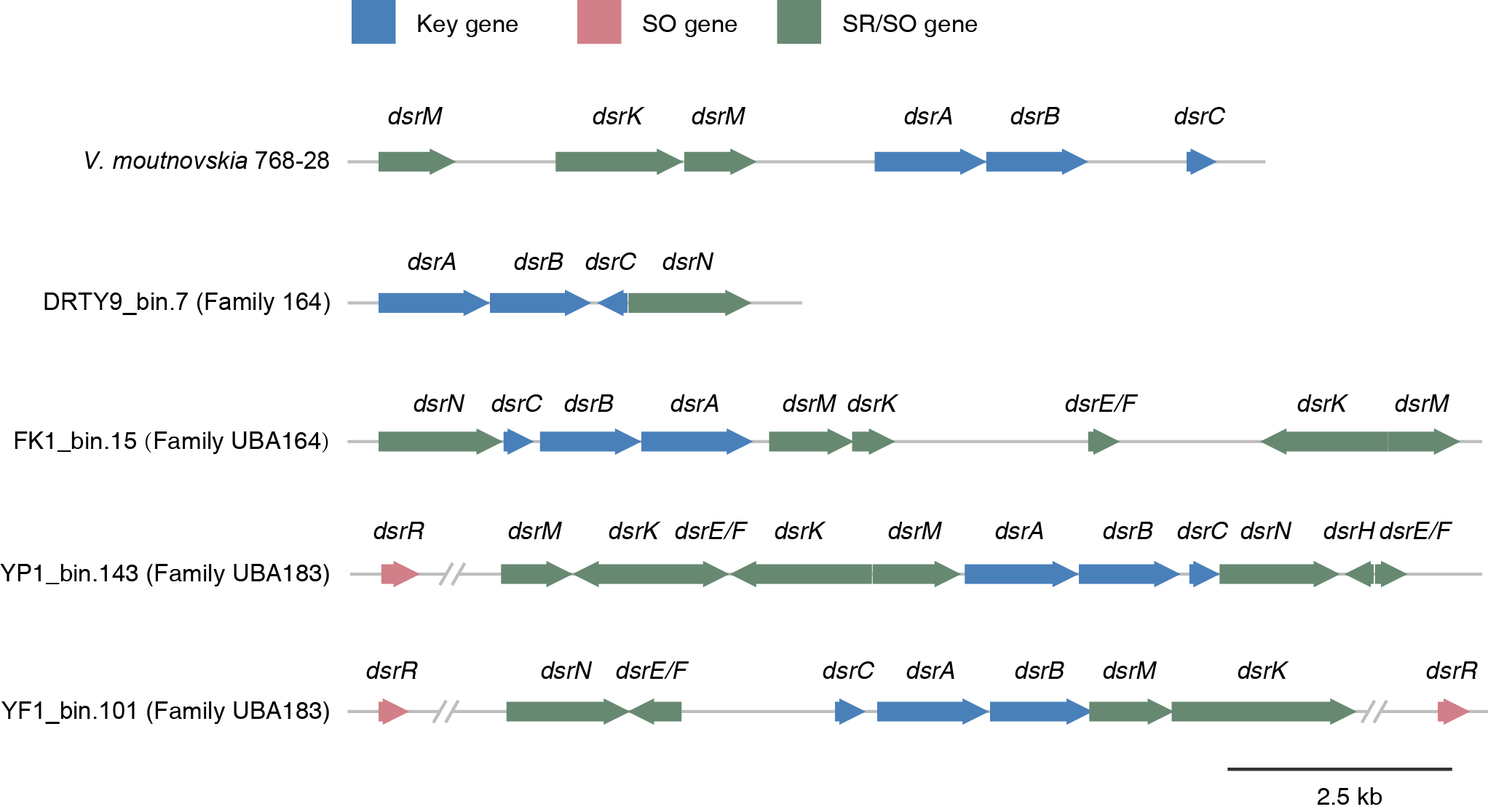


**Fig. S9** **The gene organization of *dsr* operon in *Nitrososphaeria* and *Vulcanisaeta moutnovskia* 786-28.** Colors represented different types of genes. Abbreviations: *dsrABC*, dissimilatory sulfite reductase; *dsrE*/*F/H* sulphur relay protein; *dsrK*, (Fe-S)-binding protein; *dsrM*, nitrate reductase gamma subunit; *dsrN*, cobyrinic acid a,c-diamide synthase; *dsrR*, adhesin/iron-sulfur cluster assembly protein.


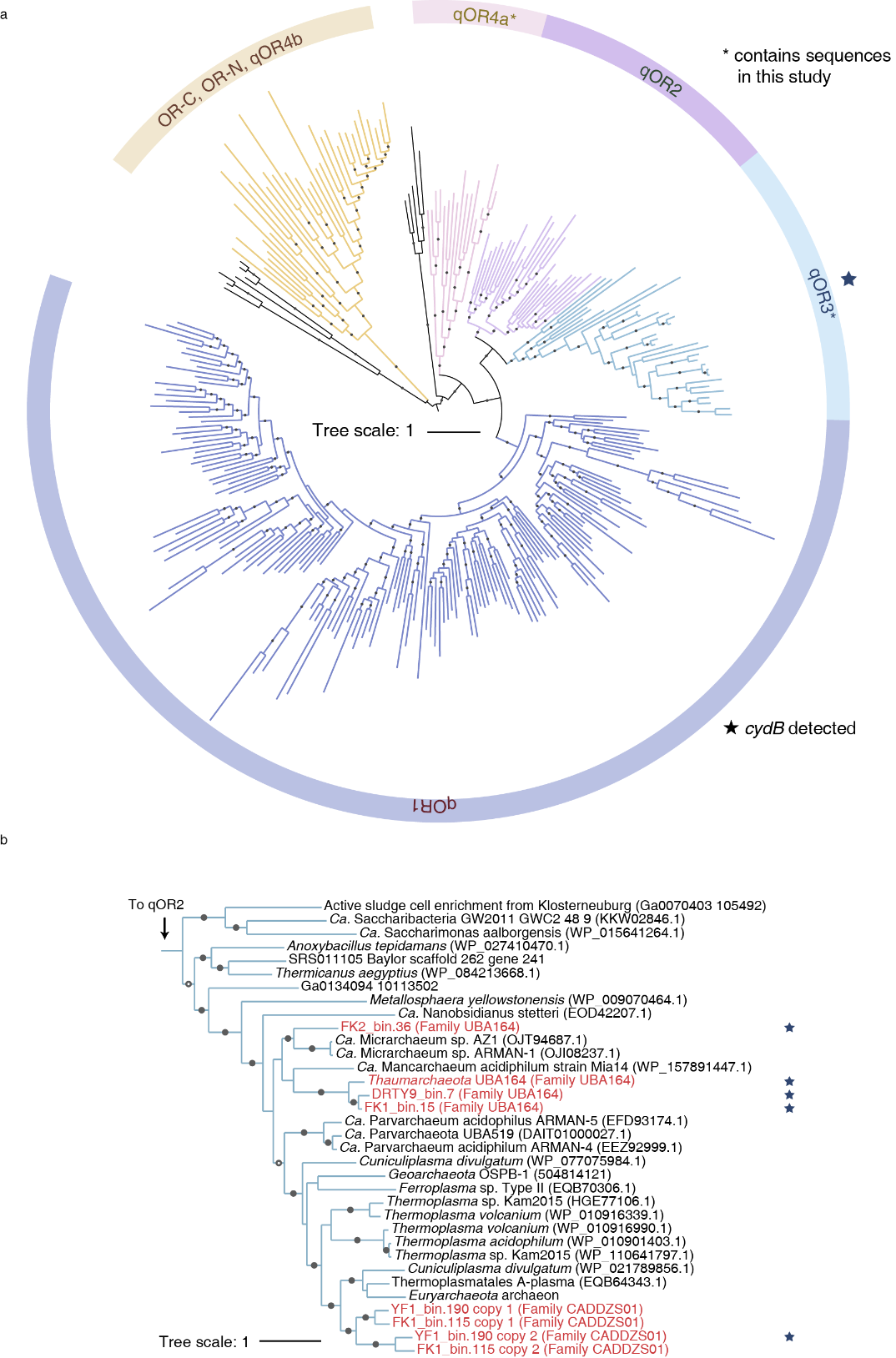


**Fig. S10 Maximum-likelihood phylogenetic tree of the homologs of CydA.** Amino acid sequences from the tree were from Murali et al. [10] and obtained from the NCBI database. All sequences were aligned with MUSCLE [3] and the alignments were filtered with TrimAL [4], resulting in an alignment length of 916 columns. The phylogeny was inferred with IQ-TREE [5] and VT+F+R9 was chosen as the best substitution model. (a) Clusters with the presence of *Nitrososphaeria* sequences were labeled with asterisks and MAGs with both *cydA* and *cydB* detected were marked with blue star (b) The enlarged phylogeny of qOR3 clade from (a). Sequences from *Nitrososphaeria* were labeled in red. Nodes with ultrafast bootstrap values ≥ 80% (60%) were indicated as solid (hollow) circles.


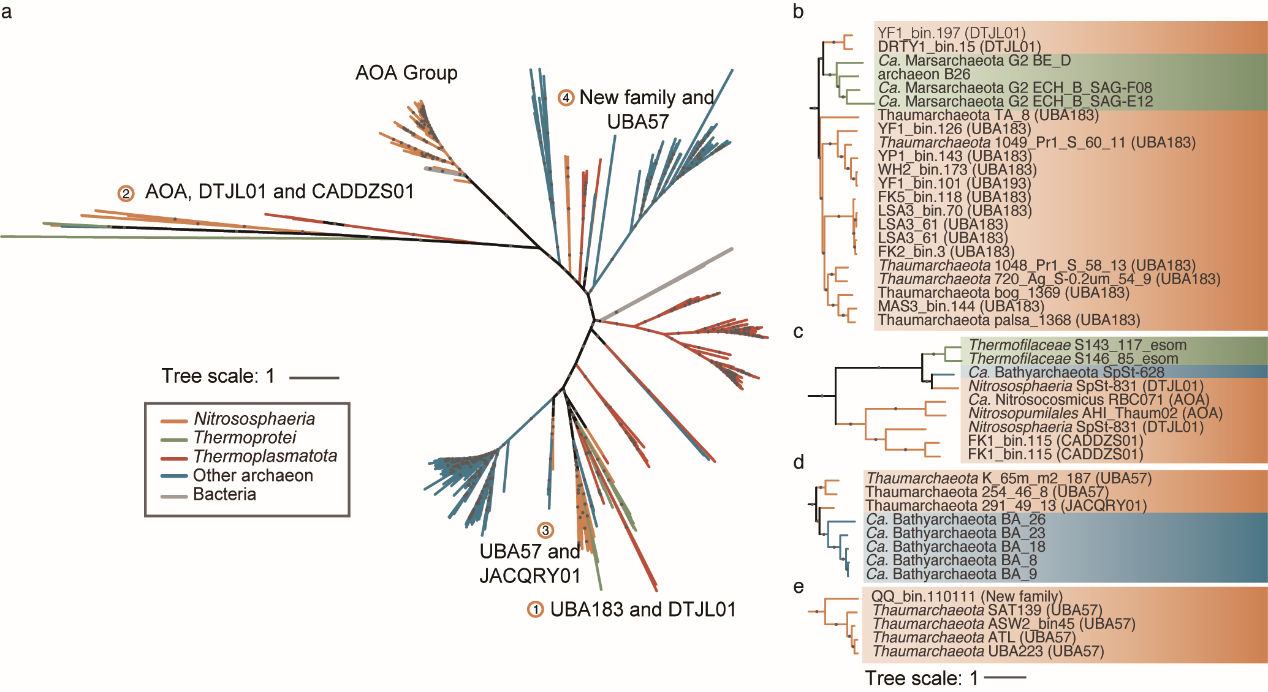


**Fig. S11 Maximum-likelihood phylogenetic tree of the homologs of CoxAB.** Amino acid sequences from the tree were obtained by querying the CoxA and CoxB hmm files against the NCBI GenBank Database. All sequences were aligned with MUSCLE [3] and the alignments were filtered with TrimAL [4], resulting in an alignment length of 1, 997 columns. The phylogeny was inferred with IQ-TREE [5] and VT+F+R10 was chosen as the best substitution model. Clusters with the presence of *Nitrososphaeria* sequences were labeled with red circles. (b-e) The enlarged phylogenies of CoxAB marked in red circles with number 1-4 in (a). Nodes with ultrafast bootstrap values ≥ 60% were indicated as solid circles.


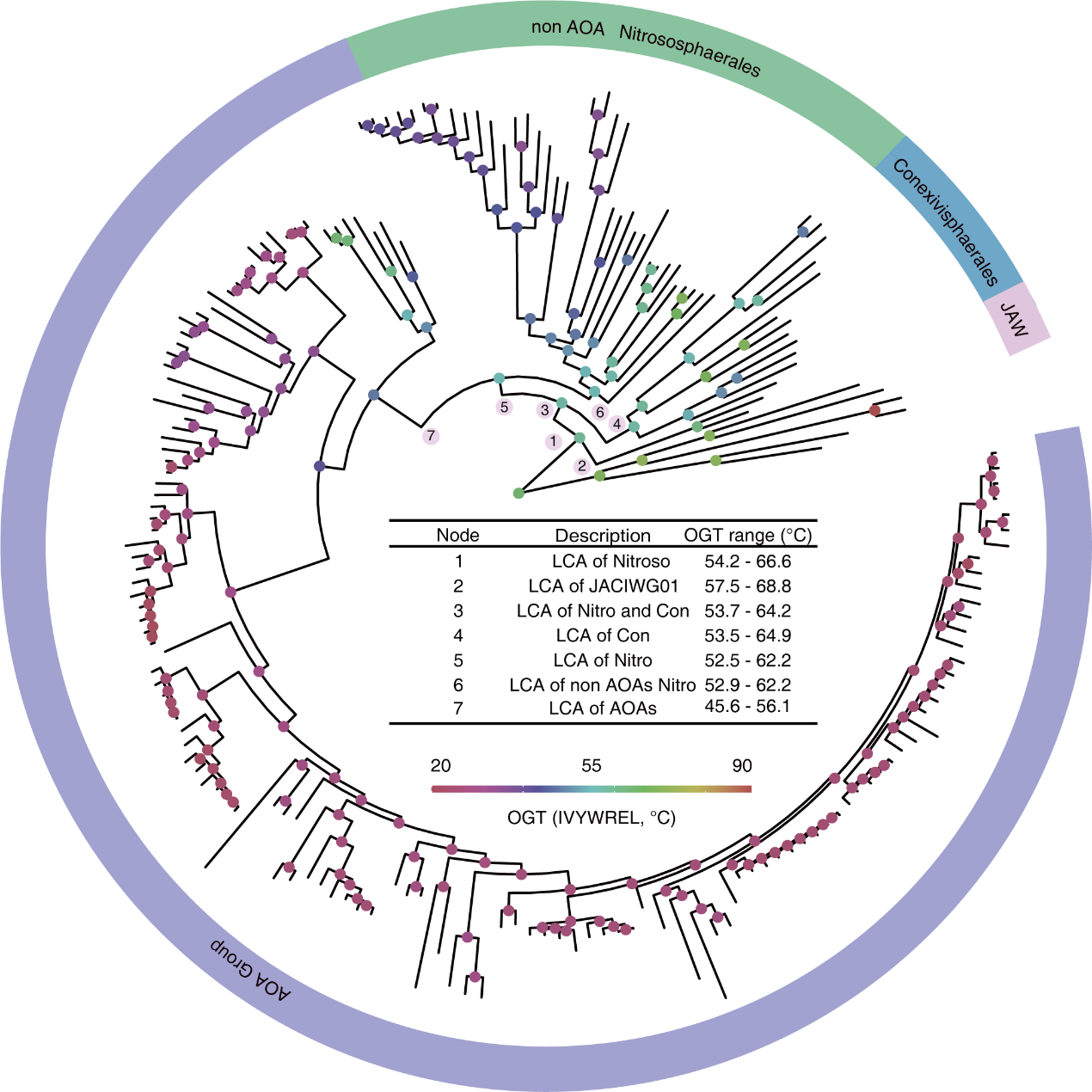


**Fig. S12** **Inference of ancestral predicted optimal growth temperature (OGT) of *Nitrososphaeria*.** The evolutionary changes of OGT predicted by IVYWREL from the common ancestor of *Nitrososphaeria* were shown. The phylogenomic tree was the same as in Fig 4. The nodes of the inferred last common ancestors were marked with pink solid circles. JAW, JAWICG01; LCA, the last common ancestor; Nitroso, *Nitrososphaeria*; Nitro, *Nitrososphaerales*; Con, *Conexivisphaerales*. The color bar in the middle indicated the range corresponding to the values of OGT.


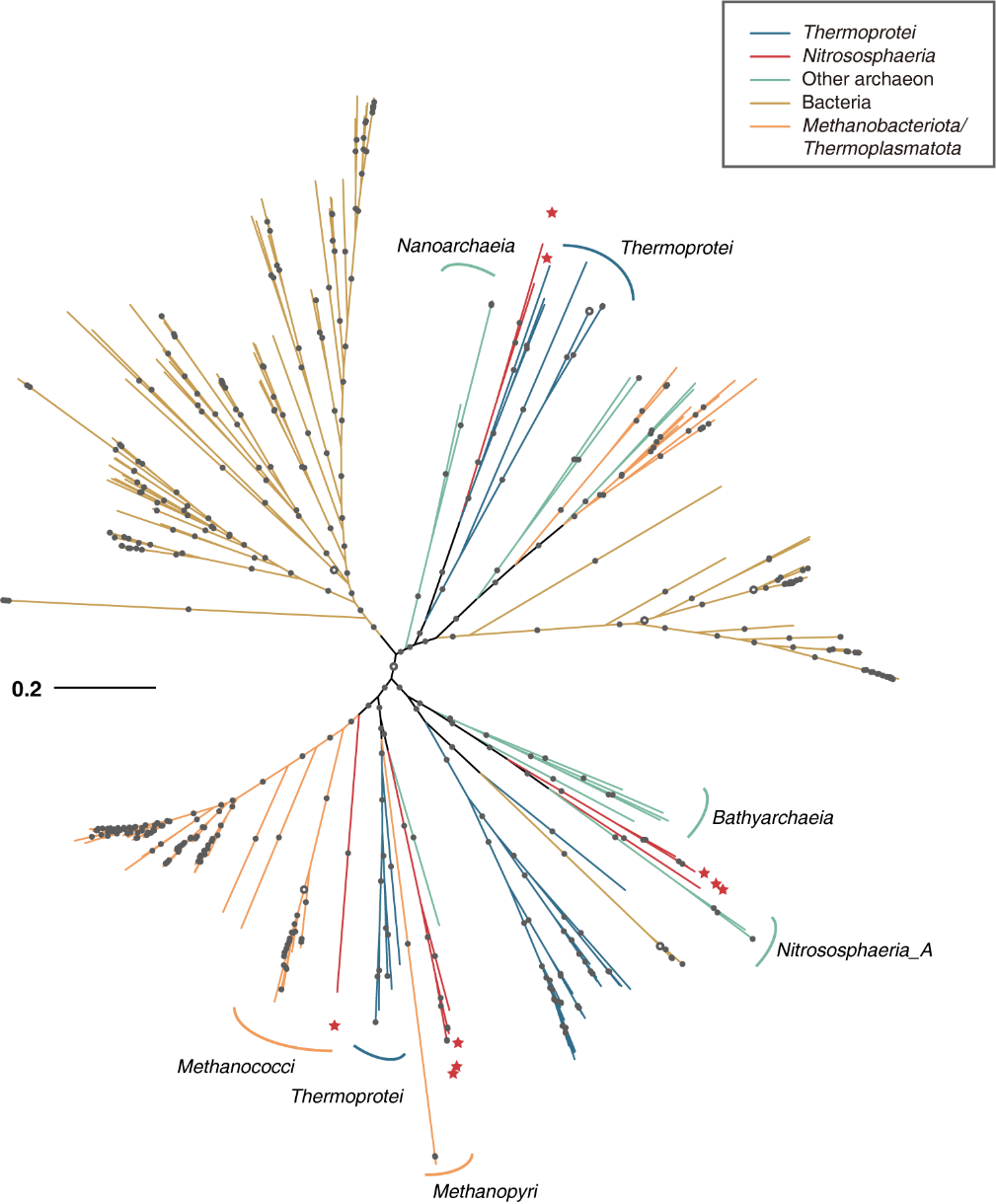


**Fig. S13** **Maximum-likelihood phylogenetic tree of the homologs of Rgy.** Amino acid sequences from the tree were obtained from Catchpole et al. [11]. All sequences were aligned with MAFFT [9] and the alignments were filtered with BMGE v.1.12 [12], resulting in an alignment length of 849 columns. The tree topology was determined using MrBayes v.3.2.7 [13] with parameters (ngen=10000000 Nruns=2 Nchains=4 diagnfreq=100000 relburnin=yes burninfrac=0.25 samplefreq=10000 printfreq=10000). The sequences from *Nitrososphaeria* were marked with red stars. Nodes with ultrafast bootstrap values ≥ 80% (60%) were indicated as solid (hollow) circles.


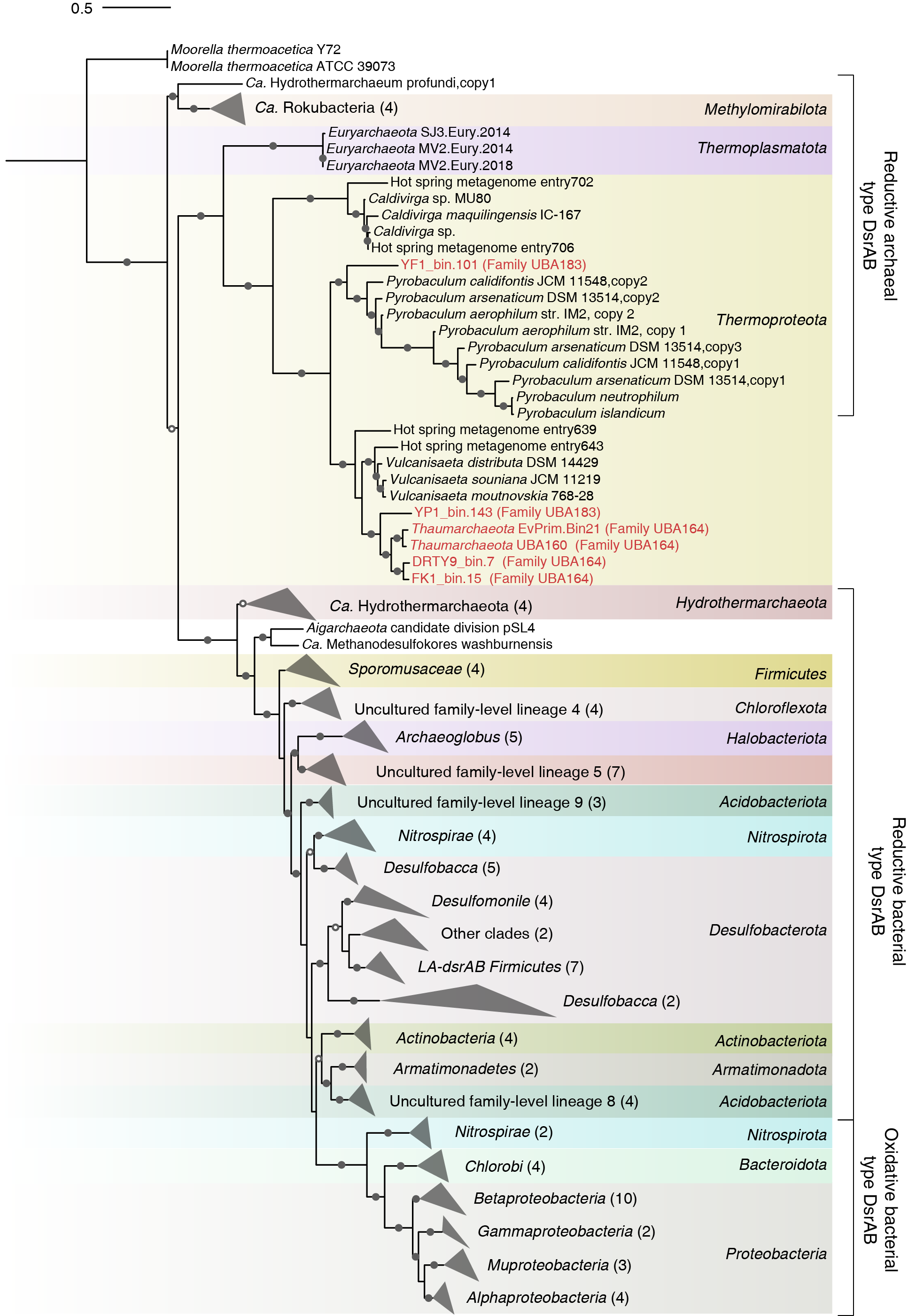


**Fig. S14** **Maximum-likelihood phylogenetic tree of the homologs of DsrAB.** Amino acid sequences from the tree were from Müller et al. [14] and obtained from the NCBI database. All sequences were aligned with MUSCLE [3] and the alignments were filtered with TrimAL [4], resulting in an alignment length of 511 columns. The phylogeny was inferred with IQ-TREE [5] and LG+I+G4 was chosen as the best substitution model. Sequences from *Nitrososphaeria* were labeled in red. Nodes with ultrafast bootstrap values ≥ 80% (60%) were indicated as solid (hollow) circles.


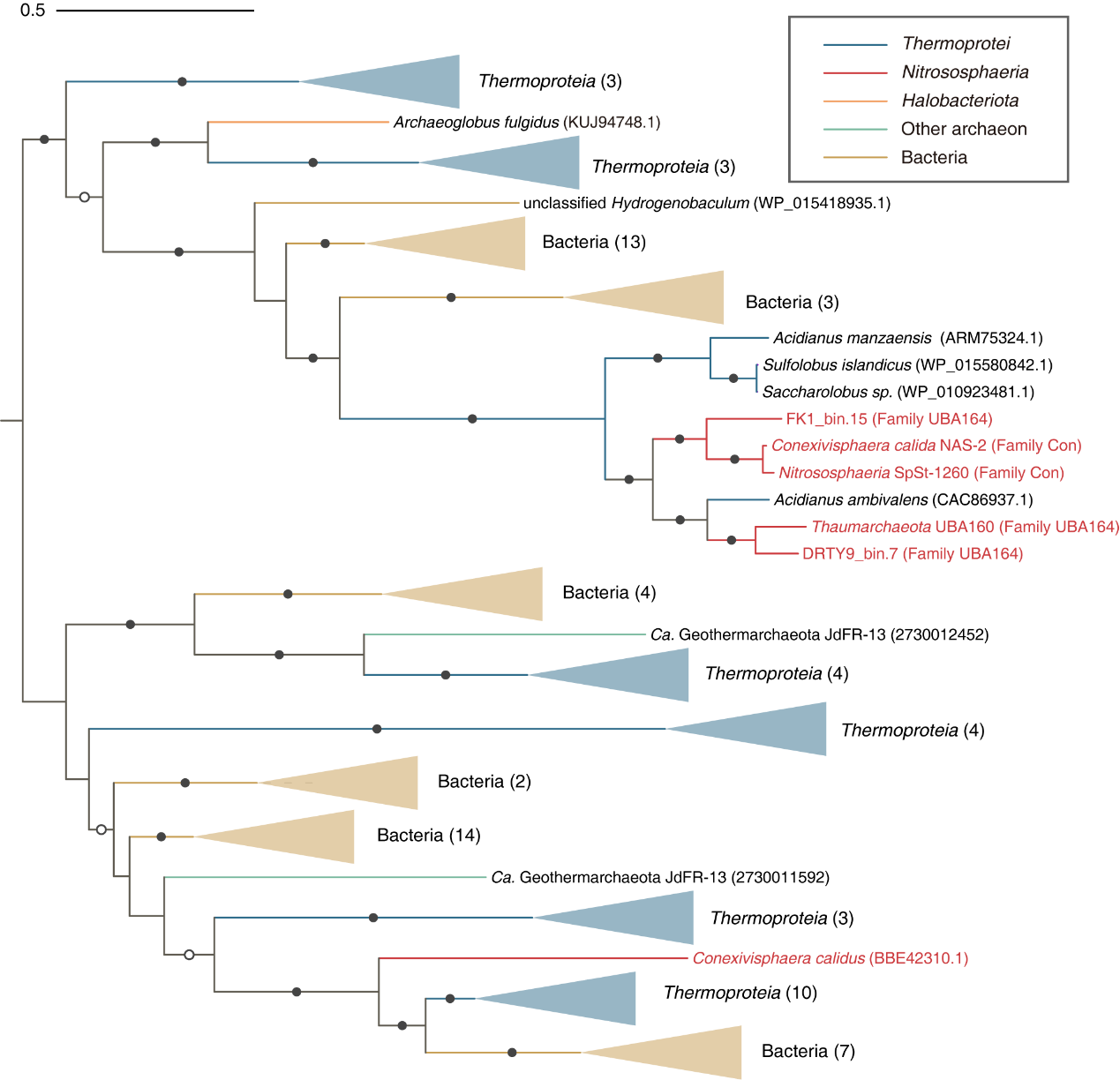


**Fig. S15 Maximum-likelihood phylogenetic tree of the homologs of Sre (upper) and Phs/Psr (lower).** Amino acid sequences from the tree were from Kato et al. [15] and obtained from the NCBI database. All sequences were aligned with MUSCLE [3] and the alignments were filtered with TrimAL [4], resulting in an alignment length of 726 columns. The phylogeny was inferred with IQ-TREE [5] and LG+F+R6 was chosen as the best substitution model. Sequences from *Nitrososphaeria* were labeled in red. Nodes with ultrafast bootstrap values ≥ 80% (60%) were indicated as solid (hollow) circles.


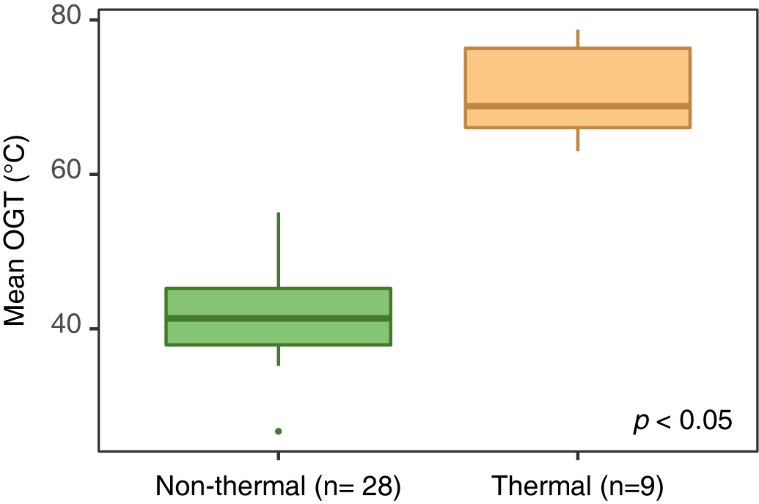


**Fig. S16 The comparison of the mean predicted growth temperatures (OGTs).** The boxplot showed the mean OGT of members inhabited the thermal habitats (yellow) and non-thermal habitats (green). The P value was obtained from the Wilcoxon rank sum test.


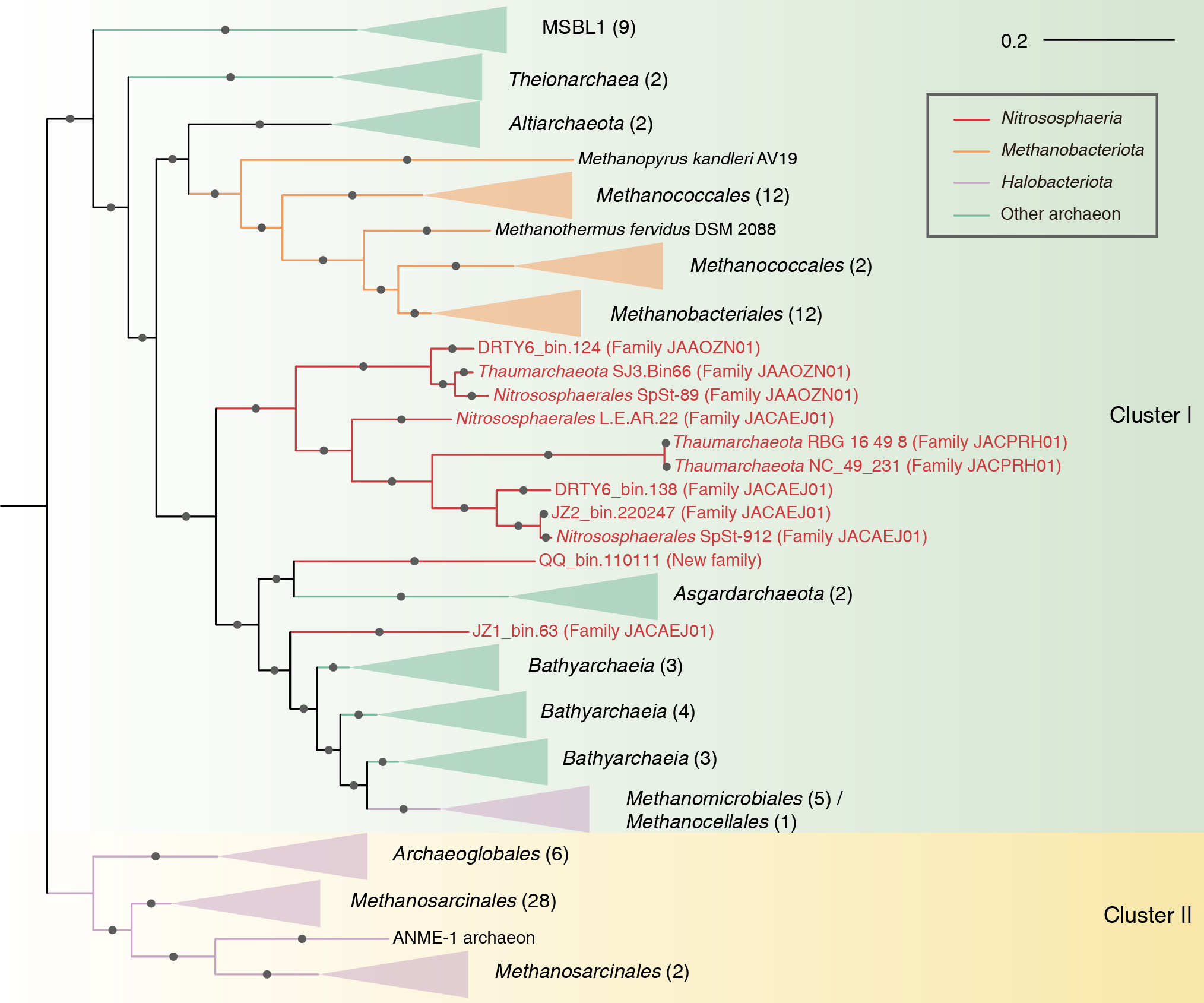


**Fig. S17 Maximum-likelihood phylogenetic tree of the homologs of CdhABCDE.** Amino acid sequences from the tree were from Adam et al. [16]. All sequences were aligned with MUSCLE [3] and the alignments were filtered with TrimAL [4], resulting in an alignment length of 2, 572 columns. The tree topology was determined using MrBayes v.3.2.7 [13] as mentioned above. The protein sequences from *Nitrososphaeria* were marked with red. Nodes with ultrafast bootstrap values ≥ 80% were indicated as solid circles.


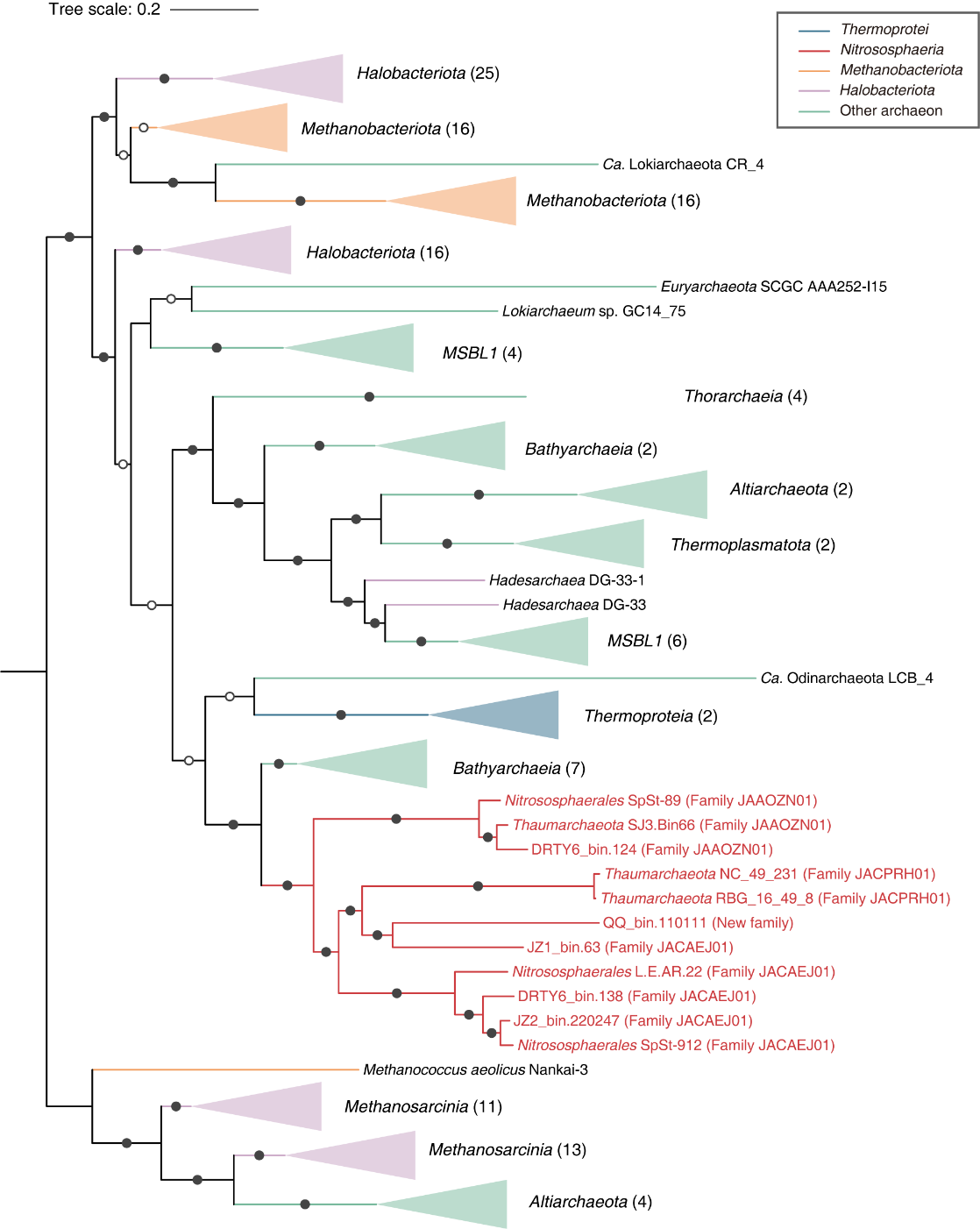


**Fig. S18 Maximum-likelihood phylogenetic tree of the homologs of FwdABCD.** Amino acid sequences from the tree were from Adam et al. [16]. All sequences were aligned with MUSCLE [3] and the alignments were filtered with TrimAL [4], resulting in an alignment length of 1, 935 columns. The phylogeny was inferred with IQ-TREE [5] and VT+F+R8 was chosen as the best substitution model. The sequences from *Nitrososphaeria* were marked with red. Nodes with ultrafast bootstrap values ≥ 80% (60%) were indicated as solid (hollow) circles.

**
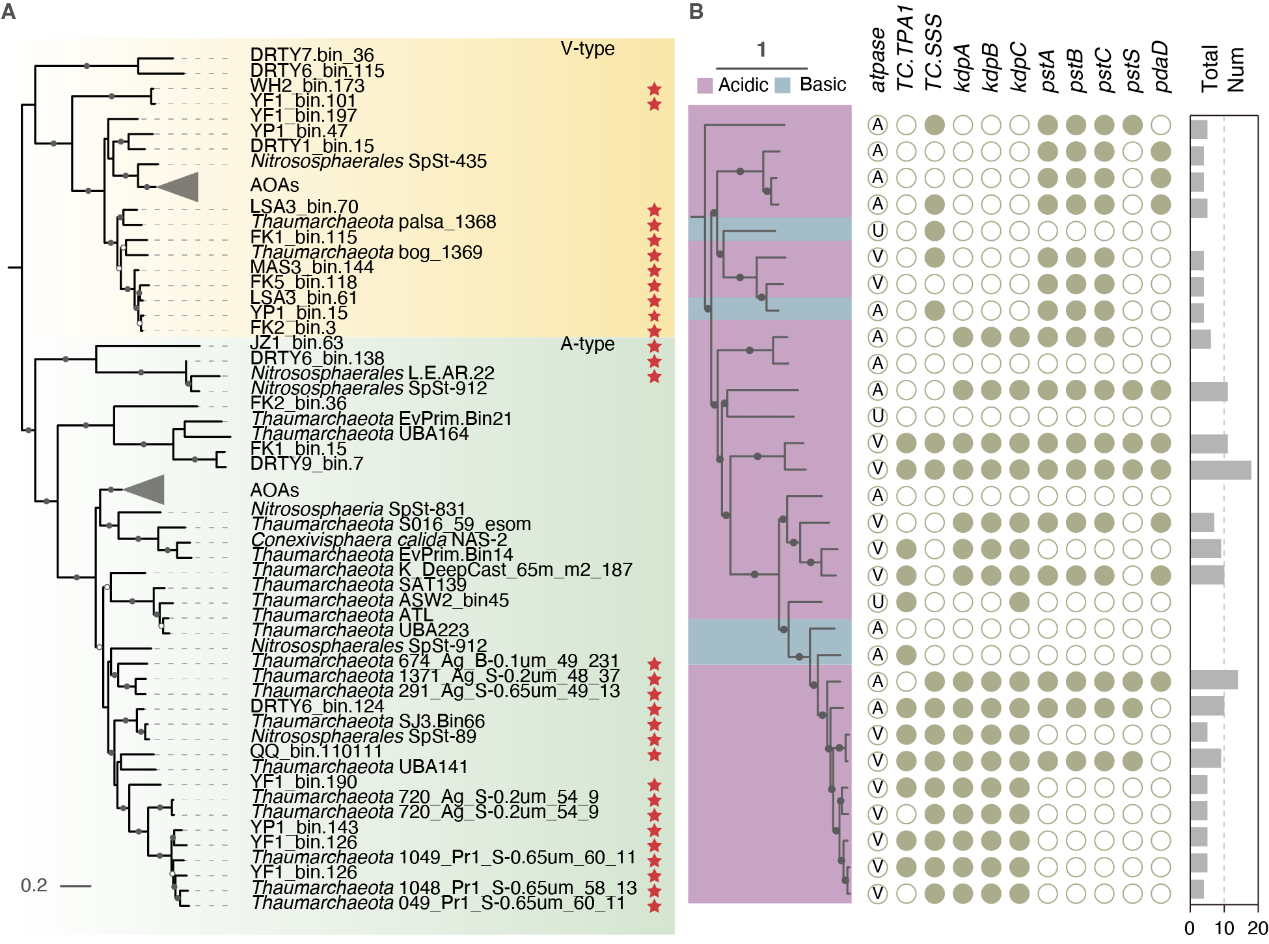
Fig. S19 Comparisons between Non-AOA *Nitrososphaerales* obtained from acidic and basic habitats.** (a) Maximum-likelihood phylogenetic tree of the homologs of AtpA. Amino acid sequences from the tree were obtained by querying the AtpA hmm file against the *Nitrososphaeria* species representatives. All sequences were aligned with MAFFT [9] and the alignments were filtered with TrimAL [4], resulting in an alignment length of 615 columns. The phylogeny was inferred with IQ-TREE [5] and Q.yeast+R6 was chosen as the best substitution model. The sequences from Non-AOA *Nitrososphaerales* were marked with red. Nodes with ultrafast bootstrap values ≥ 80% (60%) were indicated as solid (hollow) circles. The comparison of genes of interest between members from acidic and basic habitats. Abbreviations: A, A-type ATPase; V, V-type ATPase; U, unknown-type ATPase.

**Supplementary Tables**

**Supplementary Table 1.** The geographical parameters and sampling dates of the AMD sediment samples.

| **Sample** | **Location** | **Province** | **Sampling Date** | **Latitude(N)** | **Longitude(E)** |
| --- | --- | --- | --- | --- | --- |
| LSA1 | Longshenao | Guangxi | Aug-17 | 24.833685 | 107.56887 |
| LSA2 | Longshenao | Guangxi | Aug-17 | 24.833985 | 107.56856 |
| LSA3 | Longshenao | Guangxi | Aug-17 | 24.834285 | 107.56987 |
| LSA4 | Longshenao | Guangxi | Aug-17 | 24.833112 | 107.56905 |
| LSA5 | Longshenao | Guangxi | Aug-17 | 24.834976 | 107.56874 |
| WY4 | Wuyi | Guangxi | Aug-17 | 25.148969 | 107.50062 |
| DBS1-3 | Dabaoshan | Guangdong | Sep-17 | 24.55954 | 113.74507 |
| DBS2-1 | Dabaoshan | Guangdong | Sep-17 | 24.552045 | 113.7455 |
| DBS2-4 | Dabaoshan | Guangdong | Sep-17 | 24.551902 | 113.7451 |
| DBS2-5 | Dabaoshan | Guangdong | Sep-17 | 24.552367 | 113.74558 |
| FK1 | Fankou | Guangdong | Sep-17 | 25.049027 | 113.66335 |
| FK2 | Fankou | Guangdong | Sep-17 | 25.049023 | 113.66401 |
| FK3 | Fankou | Guangdong | Sep-17 | 25.049111 | 113.66402 |
| FK4 | Fankou | Guangdong | Sep-17 | 25.049037 | 113.66358 |
| FK5 | Fankou | Guangdong | Sep-17 | 25.049789 | 113.66339 |
| YF1 | Yunfu | Guangdong | Sep-17 | 22.964803 | 112.01443 |
| YF2 | Yunfu | Guangdong | Sep-17 | 22.964133 | 112.01483 |
| YF3 | Yunfu | Guangdong | Sep-17 | 22.964429 | 112.01401 |
| YF4 | Yunfu | Guangdong | Sep-17 | 22.965132 | 112.01456 |
| YF5 | Yunfu | Guangdong | Sep-17 | 22.964003 | 112.015 |
| DX2 | Dexing | Jiangxi | Sep-17 | 29.002054 | 117.72761 |
| YP1 | Yongping | Jiangxi | Sep-17 | 28.19111 | 117.76694 |
| YP2 | Yongping | Jiangxi | Sep-17 | 28.191213 | 117.76715 |
| YP3 | Yongping | Jiangxi | Sep-17 | 28.191671 | 117.76679 |
| YP4 | Yongping | Jiangxi | Sep-17 | 28.191444 | 117.7668 |
| YP5 | Yongping | Jiangxi | Sep-17 | 28.191044 | 117.76723 |
| YS3 | Yinshan | Jiangxi | Sep-17 | 28.977137 | 117.6018 |
| YS4 | Yinshan | Jiangxi | Sep-17 | 28.977012 | 117.60167 |
| YS5 | Yinshan | Jiangxi | Sep-17 | 28.977249 | 117.601 |
| MAS1 | Maanshan | Anhui | Oct-17 | 31.677216 | 118.62743 |
| MAS2 | Maanshan | Anhui | Oct-17 | 31.677977 | 118.62756 |
| MAS3 | Maanshan | Anhui | Oct-17 | 31.677766 | 118.62795 |
| MAS4 | Maanshan | Anhui | Oct-17 | 31.67721 | 118.62743 |
| MAS5 | Maanshan | Anhui | Oct-17 | 31.678001 | 118.62801 |
| TL1-5 | Tongling | Anhui | Oct-17 | 30.940541 | 117.98997 |
| WH2 | Wuhu | Anhui | Oct-17 | 31.082771 | 118.2565 |

**Supplementary Table 2.** The physicochemical parameters of AMD sediment samples.

| **Sample** | **pH** | **^a^EC** | **^b^TN** | **^b^TP** | **^b^TC** | **^b^SO_4_^2-^** | **^b^Fe^2+^** | **^b^Fe^3+^** |
| --- | --- | --- | --- | --- | --- | --- | --- | --- |
| LSA1 | 2.53 | 3.56 | 1.18 | 9.36 | 131.42 | 7.3 | 0.13 | 1.87 |
| LSA2 | 2.54 | 3.08 | 1.2 | 9.73 | 75.76 | 6.31 | 0.11 | 1.63 |
| LSA3 | 2.51 | 3.3 | 1.6 | 9.98 | 71.31 | 6.38 | 0.15 | 1.8 |
| LSA4 | 2.53 | 3.32 | 1.56 | 9.98 | 161.06 | 7.39 | 0.1 | 1.45 |
| LSA5 | 2.56 | 3.23 | 1.5 | 10.21 | 89.53 | 6.45 | 0.11 | 1.59 |
| WY4 | 4.37 | 1.89 | 0.41 | 1.31 | 37.61 | 26.2 | 0.38 | 23.71 |
| DBS1-3 | 1.87 | 1.22 | 0.2 | 0.6 | 35.3 | 5.63 | 0.09 | 2.69 |
| DBS2-1 | 2.42 | 0.38 | 0.3 | 0.6 | 4.21 | 14.19 | 0.08 | 0.13 |
| DBS2-4 | 2.46 | 0.4 | 0.06 | 0.62 | 5.05 | 13.53 | 0.05 | 0.25 |
| DBS2-5 | 2.69 | 0.42 | 0.08 | 0.52 | 6.79 | 13.72 | 0.09 | 1.3 |
| FK1 | 3.06 | 2.9 | 0.08 | 0.62 | 35.79 | 15.74 | 0.91 | 0.09 |
| FK2 | 2.49 | 2.16 | 1.02 | 3.06 | 81.95 | 18.17 | 0.05 | 1.09 |
| FK3 | 5.45 | 2.88 | 0.05 | 2.15 | 52.27 | 15.78 | 1.34 | 3.63 |
| FK4 | 2.99 | 3.1 | 0.05 | 1.94 | 32.44 | 14.58 | 0.75 | 0.1 |
| FK5 | 2.61 | 3.24 | 0.08 | 1.61 | 29.12 | 17.1 | 0.95 | 0.14 |
| YF1 | 2.69 | 1.91 | 0.37 | 2.11 | 54.11 | 6.33 | 0.88 | 33.14 |
| YF2 | 2.53 | 3.01 | 0.13 | 0.67 | 36.84 | 6.72 | 0.5 | 3.94 |
| YF3 | 2.82 | 1.67 | 0.04 | 0.4 | 62.21 | 6.97 | 0.84 | 1.12 |
| YF4 | 2.57 | 3.11 | 0.38 | 0.46 | 54.9 | 8.08 | 0.66 | 2.37 |
| YF5 | 2.89 | 1.73 | 0.04 | 0.32 | 61.52 | 5.23 | 1.22 | 0.22 |
| DX2 | 2.79 | 8.09 | 2.94 | 14.54 | 64.6 | 23.15 | 0.09 | 2.88 |
| YP1 | 2.72 | 2.55 | 0.48 | 2.08 | 18.98 | 28.46 | 0.14 | 4.78 |
| YP2 | 2.79 | 1.94 | 0.34 | 1.45 | 11.83 | 36.9 | 0.1 | 4.75 |
| YP3 | 2.82 | 1.91 | 0.36 | 2.01 | 23.86 | 37.65 | 0.17 | 4.99 |
| YP4 | 2.93 | 2.51 | 0.42 | 1.88 | 18.05 | 53.37 | 0.16 | 4.09 |
| YP5 | 2.94 | 1.51 | 0.24 | 1.55 | 16.78 | 36.13 | 0.23 | 4.93 |
| YS3 | 2.75 | 2.38 | 1.01 | 2.49 | 32.57 | 12.9 | 0.58 | 31.33 |
| YS4 | 2.82 | 2.53 | 0.95 | 2.43 | 31.87 | 10.3 | 0.61 | 31.49 |
| YS5 | 2.95 | 1.79 | 0.94 | 2.35 | 18.12 | 7.1 | 0.51 | 26.92 |
| MAS1 | 2.62 | 3.38 | 0.43 | 3.05 | 29.15 | 4.83 | 0.08 | 0.75 |
| MAS2 | 2.8 | 3.68 | 0.4 | 4.19 | 24.37 | 5.19 | 0.09 | 0.69 |
| MAS3 | 2.58 | 4.3 | 0.57 | 5.38 | 28.34 | 9.56 | 0.13 | 1.39 |
| MAS4 | 2.61 | 3.75 | 0.12 | 3.04 | 33.74 | 5.68 | 0.05 | 0.62 |
| MAS5 | 2.67 | 3.11 | 1.55 | 2.78 | 42.76 | 5.92 | 0.06 | 0.7 |
| TL1-5 | 2.5 | 4.48 | 0.15 | 1.57 | 110.3 | 16.99 | 0.04 | 0.72 |
| WH2 | 2.96 | 3.04 | 0.05 | 2.7 | 67.24 | 8.99 | 0.4 | 5.69 |

**a** unit: ms cm-1

**b** unit: g kg-1

**Supplementary Table 3.** List of the ribosomal proteins selected for phylogenomic analyses.

| arCOG_ID | Gene | Annotation |
| --- | --- | --- |
| arCOG00779 | RplO | Ribosomal protein L15 |
| arCOG00780 | RPL18A | Ribosomal protein L18E |
| arCOG00781 | RPL32 | Ribosomal protein L32E |
| arCOG00785 | RpmC | Ribosomal protein L29 |
| arCOG01344 | RPS19A | Ribosomal protein S19E (S16A) |
| arCOG01722 | RpsM | Ribosomal protein S13 |
| arCOG01751 | Rpl7Ae | Ribosomal protein L7AE |
| arCOG01752 | RPL30 | Ribosomal protein L30E |
| arCOG01758 | RpsJ | Ribosomal protein S10 |
| arCOG01885 | RPS17A | Ribosomal protein S17E |
| arCOG01946 | RPS6A | Ribosomal protein S6E/S10 |
| arCOG01950 | RPL24A | Ribosomal protein L24E |
| arCOG04067 | RplB | Ribosomal protein L2 |
| arCOG04070 | RplC | Ribosomal protein L3 |
| arCOG04071 | RplD | Ribosomal protein L4 |
| arCOG04072 | RplW | Ribosomal protein L23 |
| arCOG04086 | RpmD | Ribosomal protein L30 |
| arCOG04087 | RpsE | Ribosomal protein S5 |
| arCOG04088 | RplR | Ribosomal protein L18 |
| arCOG04089 | RPL19A | Ribosomal protein L19E |
| arCOG04090 | RplF | Ribosomal protein L6P |
| arCOG04091 | RpsH | Ribosomal protein S8 |
| arCOG04092 | RplE | Ribosomal protein L5 |
| arCOG04093 | RPS4A | Ribosomal protein S4E |
| arCOG04094 | RplX | Ribosomal protein L24 |
| arCOG04095 | RplN | Ribosomal protein L14 |
| arCOG04096 | RpsQ | Ribosomal protein S17 |
| arCOG04097 | RpsC | Ribosomal protein S3 |
| arCOG04098 | RplV | Ribosomal protein L22 |
| arCOG04099 | RpsS | Ribosomal protein S19 |
| arCOG04108 | RPS27A | Ribosomal protein S27E |
| arCOG04109 | RPL42A | Ribosomal protein L44E |
| arCOG04113 | RplP | Ribosomal protein L10AE/L16 |
| arCOG04129 | RPL21A | Ribosomal protein L21E |
| arCOG04154 | RPS8A | Ribosomal protein S8E |
| arCOG04183 | RPS27AE | Ribosomal protein S27AE |
| arCOG04185 | RpsO | Ribosomal protein S15P |
| arCOG04186 | RPS1A | Ribosomal protein S3AE |
| arCOG04208 | RPL43A | Ribosomal protein L37AE/L43A |
| arCOG04209 | RPL15A | Ribosomal protein L15E |
| arCOG04239 | RpsD | Ribosomal protein S4 or related protein |

|  | |  | | (continued Supplementary Table 3) | |
| --- | --- | --- | --- | --- | --- |
| arCOG_ID | Gene | | Annotation | |  |
| arCOG04240 | | RpsK | | Ribosomal protein S11 | |
| arCOG04242 | | RplM | | Ribosomal protein L13 | |
| arCOG04243 | | RpsI | | Ribosomal protein S9 | |
| arCOG04245 | | RpsB | | Ribosomal protein S2 | |
| arCOG04254 | | RpsG | | Ribosomal protein S7 | |
| arCOG04255 | | RpsL | | Ribosomal protein S12 | |
| arCOG04287 | | RPP1A | | Ribosomal protein L12E/L44/L45/RPP1/RPP2 | |
| arCOG04288 | | RplJ | | Ribosomal protein L10 | |
| arCOG04289 | | RplA | | Ribosomal protein L1 | |
| arCOG04305 | | RPS26B | | Ribosomal protein S26 | |
| arCOG04314 | | RPS28A | | Ribosomal protein S28E/S33 | |
| arCOG04372 | | RplK | | Ribosomal protein L11 | |
| arCOG04473 | | RPL31A | | Ribosomal protein L31E | |

**Supplementary Data 1.** The basic genomic features of publicly available *Nitrososphaeria* genomes**.**

**Supplementary Data 2.** List of genes in *Nitrososphaeria* and featured in Fig. 2 and Fig. S4.

**Supplementary Data 3.** The basic genomic features of the Nitrososphaeria MAGs reconstructed in the present study.

**Supplementary Data 4.** The presence of non-AOA *Nitrososphaeria* in publicly available amplicon-based sequencing data.

**Supplementary Data 5.** The sequence identities between *dsrAB* genes in *Nitrososphaeria* and those in *Thermoprotei*.

**References**

1. King GM. Molecular and culture-based analyses of aerobic carbon monoxide oxidizer diversity. *Appl Environ Microbiol.* 2003;69:7257–7265.
2. King GM, Weber CF. Distribution, diversity and ecology of aerobic CO-oxidizing bacteria. *Nat Rev Microbiol.* 2007;5:107–118.
3. Edgar RC. MUSCLE: a multiple sequence alignment method with reduced time and space complexity. *BMC Bioinformatics.* 2004;5:113.
4. Capella-Gutiérrez S, Silla-Martínez JM, Gabaldón T. trimAl: a tool for automated alignment trimming in large-scale phylogenetic analyses. *Bioinformatics.*

2009;25:1972–1973.

1. Minh BQ, Schmidt HA, Chernomor O, Schrempf D, Woodhams MD, von Haeseler A, et al. IQ-TREE 2: new models and efficient methods for phylogenetic inference in the genomic era. *Mol Biol Evol.* 2020;37:1530–1534.
2. Kitzinger K, Koch H, Lücker S, Sedlacek CJ, Herbold C, Schwarz J, et al. Characterization of the first “*Candidatus* Nitrotoga” isolate reveals metabolic versatility and separate evolution of widespread nitrite-oxidizing bacteria. *MBio.* 2018;9:e01186–18.
3. Helen D, Kim H, Tytgat B, Anne W. Highly diverse *nirK* genes comprise two major clades that harbor ammonium-producing denitrifiers. *BMC Genomics.* 2016;17:155.
4. Kerou M, Offre P, Valledor L, Abby SS, Melcher M, Nagler M, et al. Proteomics and comparative genomics of *Nitrososphaera viennensis* reveal the core genome and adaptations of archaeal ammonia oxidizers. *Proc Natl Acad Sci USA.* 2016;113:7937–7946.
5. Katoh K, Standley DM. MAFFT multiple sequence alignment software version 7: improvements in performance and usability. *Mol Biol Evol.* 2013;304:772–780.
6. Murali R, Gennis RB, Hemp J. Evolution of the cytochrome bd oxygen reductase superfamily and the function of CydAA’ in Archaea. *ISME J.* 2021;15:3534–3548.
7. Catchpole RJ, Forterre P. The evolution of reverse gyrase suggests a nonhyperthermophilic last universal common ancestor. *Mol Bio Evol.* 2019;36:2737–2747.
8. Criscuolo A, Gribaldo S. BMGE (block mapping and gathering with entropy): a new software for selection of phylogenetic informative regions from multiple sequence alignments. *BMC Evol Biol.* 2010;10:210.
9. Ronquist F, Teslenko M, van der Mark P, Ayres DL, Darling A, Höhna S, et al. MrBayes 3.2: efficient Bayesian phylogenetic inference and model choice across a large model space. *Syst Biol.* 2012;613:539–542.
10. Müller AL, Kjeldsen KU, Rattei T, Pester M, Loy A. Phylogenetic and environmental diversity of DsrAB-type dissimilatory (bi) sulfite reductases. *ISME J.* 2015;9:1152–1165.
11. Kato S, Itoh T, Yuki M, Nagamori M, Ohnishi M, Uematsu K, et al. Isolation and characterization of a thermophilic sulfur- and iron-reducing thaumarchaeote from a terrestrial acidic hot spring. *ISME J.* 2019;13:2465–2474.
12. Adam PS, Borrel G, Gribaldo S. Evolutionary history of carbon monoxide dehydrogenase/acetyl-CoA synthase, one of the oldest enzymatic complexes. *Proc Natl Acad Sci USA*. 2018;115:E5837.
